# Systemic immune classification related to immune exhaustion evaluates the clinical response of patients with HBV-HCC after transarterial chemoembolization

**DOI:** 10.3389/fimmu.2025.1629052

**Published:** 2025-12-19

**Authors:** Lihua Yu, Xiaoli Liu, Ying Hu, Huiwen Yan, Yuqing Xie, Zimeng Shang, Yuling Liang, Wanxin Shi, Juan Du, Yuyong Jiang, Henghui Zhang, Zhiyun Yang

**Affiliations:** 1 Center of Integrative Medicine, Beijing Ditan Hospital, Capital Medical University, Beijing, China; 2 Biomedical Innovation Center, Beijing Shijitan Hospital, Capital Medical University, Beijing, China; 3 Beijing Key Laboratory for Therapeutic Cancer Vaccines, Beijing Shijitan Hospital, Capital Medical University, Beijing, China; 4 Beijing Key Laboratory of Emerging Infectious Diseases, Institute of Infectious Diseases, Beijing Ditan Hospital, Capital Medical University, Beijing, China

**Keywords:** hepatitis B virus−related hepatocellular carcinoma, transarterial chemoembolization, immune exhaustion, systemic immune classification, prognosis

## Abstract

Transarterial chemoembolization (TACE) has the potential to activate the immune system and regulate the tumor microenvironment. This study assesses the clinical response of patients with HBV-related hepatocellular carcinoma (HBV-HCC) after TACE treatment based on systemic immune classification (SIC). A total of 80 patients with HBV-HCC were assessed for the peripheral blood immune exhaustion phenotype and immune proteins through a combination of “Olink High Sensitivity Plasma Proteomics” and “Multicolor Flow Cytometry.” An unsupervised clustering algorithm was employed to classify various immune subtypes and identify core indicators that evaluate the response of SIC to TACE treatment. The application of these two technologies as novel approaches for detecting HBV-HCC provides synergistic insights into disease mechanisms and patient prognosis. Based on the combination of immune exhaustion phenotypes and immune proteins, we developed SIC that classified the subjects into three clusters: Cluster 1, Cluster 2, and Cluster 3. Cluster 3 was associated with poor clinical characteristics, unfavorable prognosis, and elevated levels of immune checkpoint expression. This risk scoring system is capable of predicting the overall survival of patients at various time points, with receiver operating characteristic areas exceeding 0.8. This study establishes SIC method to predict the clinical response of HBV-HCC patients after TACE treatment, providing new guidance for future immunotherapy and identification of non-invasive biomarkers.

## Graphical Abstract

**Figure:**
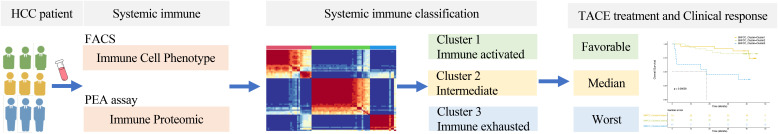

## Introduction

Hepatocellular carcinoma (HCC) continues to present a significant global health challenge, ranking as a leading cause of cancer mortality (1, 2). The hepatitis B virus (HBV) is a major etiological factor, accounting for over 50% of new HCC cases worldwide, as reported by GLOBOCAN 2022 (3). Although the global incidence of HCC is declining, the prognosis for HBV-related HCC (HBV-HCC) remains particularly poor, with a five-year survival rate of less than 20% (4). In China, where the prevalence of HBV is high, the impact of this challenge is profound; HBV-HCC constitutes approximately 80% of new cases, contributing significantly to the disease burden (5). Among the various treatment options for HCC, transarterial chemoembolization (TACE) plays a crucial and versatile role throughout the disease spectrum (6). TACE serves as the foundation for numerous comprehensive treatment regimens. Importantly, the therapeutic benefits of TACE extend beyond local cytotoxic effects. TACE-induced tumor necrosis can result in the release of tumor antigens, potentially enhancing tumor-specific immune responses and facilitating the implementation of combination therapies (7).

The impact of TACE on the immune status of patients with HCC has been extensively investigated. Studies have revealed a significant reduction in the number of CD8^+^ T cells within the tumor microenvironment following TACE treatment, accompanied by a notable increase in tumor-associated macrophages (TAM) (8). Furthermore, TACE may facilitate the conversion of CD8^+^ effector memory T cells into CD8^+^ effector T cells, thereby transforming the immunosuppressive microenvironment into one that supports immune activity (9). The CHANCE001 study, the largest multicenter real-world investigation of TACE combined with PD-(L)1 inhibitors and molecular targeted treatments (MTT) for HCC in China, demonstrated that this combination therapy significantly improved progression-free survival (PFS) (9.5 months vs. 8.0 months, P = 0.002, 95% CI, 6.6-9.5) and overall survival (OS) (19.2 months vs. 15.7 months, P = 0.001, 95% CI, 13.0-20.2) in patients with advanced HCC compared to TACE monotherapy (10). Currently, multiple phase III clinical trials are ongoing, examining the combination of TACE with targeted therapies or immunotherapies.

The current clinical evaluation of the efficacy of TACE in HCC remains heavily reliant on conventional clinical features (11, 12), radiological assessments (13, 14), and more recently, deep learning-based imaging analyses (15). While these tools provide essential structural and prognostic information, they offer limited insight into the underlying immunological mechanisms that influence treatment response. Although immune biomarkers hold considerable promise for refining prognostic stratification, the potential of non-invasive immune profiling of the peripheral blood microenvironment in predicting TACE outcomes remains largely unexplored. Existing immune classification frameworks for HCC also exhibit notable limitations in this specific therapeutic context. For instance, tumor microenvironment-based classifications typically require invasive tissue biopsies, which are seldom repeated post-TACE and fail to capture dynamic, systemic immune changes (16). Conversely, circulating immune signatures often focus on general inflammatory markers and lack specificity to T/NK cell exhaustion-a key mechanism of immunotherapy resistance that is increasingly recognized as relevant to the immunosuppressive microenvironment induced by TACE (17).

In our previous work, we established a link between exhausted CD8^+^ T and NK cell exhaustion phenotypes and unfavorable prognosis in HBV-HCC (18, 19). In light of this, we propose a novel, non-invasive systemic immune classification (SIC) aimed at addressing these deficiencies. Our approach is characterized by three critical features: (1) it utilizes exclusively peripheral blood, facilitating serial monitoring in clinical settings where repeated tissue sampling is impractical; (2) it specifically targets immune exhaustion by incorporating both cellular exhaustion phenotypes and key soluble plasma proteins (e.g., Galectin-9) that directly influence these pathways, thereby providing deeper mechanistic insights; and (3) it is explicitly designed and validated to predict outcomes following TACE therapy.

This study aimed to systematically assess the predictive value of immune exhaustion phenotypes derived from peripheral blood for the response to TACE in patients with HBV-HCC. By concentrating on peripheral immune cells and plasma proteins, we intend to develop a more comprehensive and clinically relevant profiling method. Ultimately, this non-invasive immune stratification strategy is anticipated to enhance individualized treatment sequencing, particularly in optimizing the integration of TACE with subsequent immunotherapy.

## Materials and methods

### Patient inclusion and sample collection

Approved by the Ethics Committee of Beijing Ditan Hospital, Capital Medical University, patients were enrolled and blood samples were collected for study. The included HBV-HCC patients had been registered on ClinicalTrials.gov (NCT04264962 and NCT02927626) and signed informed consent. Blood samples were processed to separate 2ml of plasma and peripheral blood mononuclear cells (PBMCs) in accordance with previously published studies and stored at -80°C (20). All plasma specimens are thawed immediately before use.

**Figure 1:**
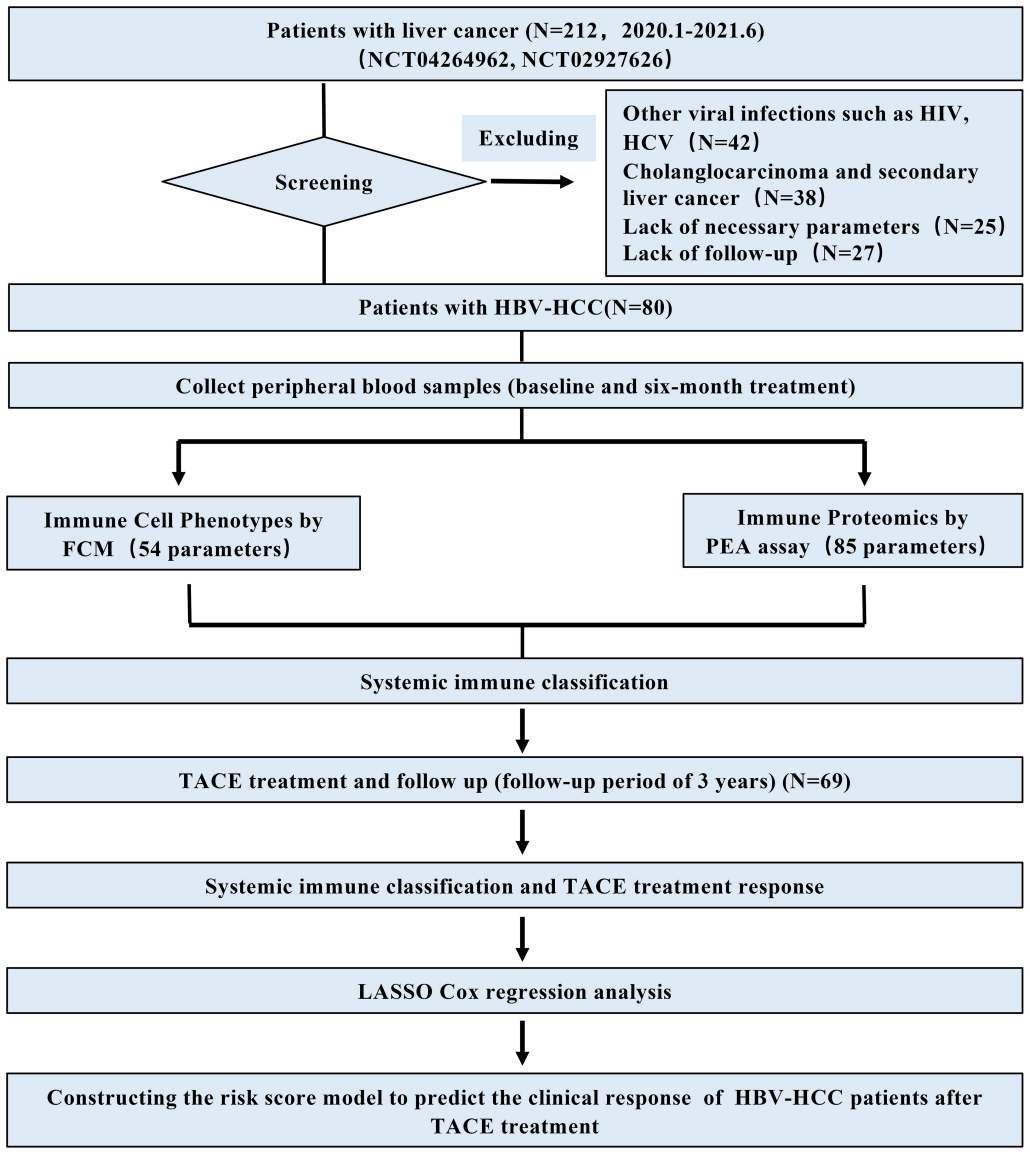
The flow chart of this study. Based on two clinical registration numbers (NCT04264962, NCT02927626), a total of 212 HCC patients were included. According to the inclusion and exclusion criteria, HBV-HCC patients (N = 80) were ultimately included. Peripheral blood samples of the HBV-HCC patients were collected for immune exhaustion phenotype and immune protein detection, and a systemic immune classification was constructed. Among them, a total of 69 patients received TACE treatment, and the predictive and prognostic value of risk scoring based on the SIC was evaluated.

The definition of HBV-HCC meets the diagnostic criteria for HCC in the Chinese guidelines and is positive for serum hepatitis B surface antigen (HBsAg) ≥6 months (21). There were initially 212 patients in this study, excluded: (1) other viral infections such as HIV, HCV (N = 42); (2) cholanglocarcinoma and secondary liver cancer (N = 38); (3) absence of necessary parameters (N = 25); (4) lack of follow-up (N = 27). Ultimately, 80 eligible HBV-HCC patients were included in this real-world study, of which 69 patients underwent TACE treatment. A research flow chart illustrating this process is presented in **Figure 1**.

### Immune exhaustion phenotype analysis

The PBMC of HBV-HCC patients was incubated with directly coupled antibodies and analyzed by multi-parametric flow cytometry. The staining antibodies were anti-human CD3-BV786 (SK7), CD4-APC-Fire750 (SK3), CD8-BV510 (SK1), CD19-APC-H7 (SJ25C1), CD14-BV650 (M5E2), CD56-BV510 (HCD56), CD16-BV711 (3G8), TIGIT-PE-CY7 (MBSA43), TIM-3-FITC (F38-2E2), PD-1-PE (EH12.2H7), CD39-APC (A1), LAG-3-AF700 (T47-530). Data collection and statistical analysis were performed using LSR Fortessa flow cytometry (BD Biosciences) and FlowJo Software.

### Immunoproteomic analysis of plasma factors

Plasma samples for this study were obtained using the Olink multiplex proximity extension assay (PEA) immuno-oncology panel, which comprises 92 proteins (Olink Bioscience AB, Sweden). The target proteins are analyzed via PEA, a double-recognition immunoassay wherein two matching antibodies simultaneously bind to a target and are labeled with a unique DNA oligonucleotide. The presence of proteins in the solution facilitates the close proximity of the two antibodies, allowing for the hybridization of their DNA oligonucleotides, which subsequently serves as a template for the DNA polymerase-dependent extension step. Following this, PCR amplification was conducted, and quantitative detection was achieved through multi-quantitative PCR, resulting in high sensitivity, specificity, and throughput in the detection of multiple protein indices. The data generated by Olink is expressed as normalized protein expression values and undergoes quality control. Among the 88 samples in this study, three quality control reports indicated warnings; however, these were classified as non-outlier samples and could be retained for further analysis. Seven of these proteins were below the lower limit of detection and were therefore excluded from subsequent analysis.

### Identification of SIC by unsupervised clustering

To identify SIC, we conducted unsupervised clustering on the protein expression data. Initially, the data were log2-transformed (using log2(x+1)) to stabilize variance, followed by Z-score normalization to scale each protein to a mean of and a standard deviation of 1. This approach ensured that all variables contributed equally to the clustering analysis. We employed hierarchical clustering with average linkage, which effectively balances intra-cluster cohesion and inter-cluster separation. To ascertain the optimal number of clusters (k), we calculated the mean silhouette width for k values ranging from 2 to 10. The highest mean silhouette width (0.78) was observed at k=3, indicating optimal cluster compactness and separation. Consequently, all patients were categorized into three distinct immune classifications.

### Immunohistochemical staining

Tumor tissue and adjacent tissues of HCC patients from were cut into 4um thick paraffin sections and stained immunohistochemically with Galectin-9 (Cat# 54330, CST) antibody. All staining procedures were carried out according to the manufacturer’s instructions. All the evaluations were done by the same pathologist. The sections were scanned by panoramic scanning electron microscope, and the positive staining was analyzed by ImageJ software (version 1.8.0).

### Statistical analysis

Unsupervised clustering was performed to identify subtypes based on the integrated profile of immune exhaustion phenotypes and immune proteins. All statistical analyses were conducted using R software and GraphPad Prism. Feature selection was performed using least absolute shrinkage and selection operator (LASSO) Cox regression. The optimal tuning parameter (λ) was determined through 10-fold cross-validation, with the λ value that yielded the minimum binomial deviance (lambda.min) being selected to identify the most predictive features while preventing overfitting. This process resulted in the selection of five core indicators for the final riskScore model. To compare of continuous variables across the three clusters, one-way ANOVA was utilized for normally distributed data, followed by *post-hoc* testing with Bonferroni correction for multiple comparisons. For non-normally distributed data, the Kruskal-Wallis test was used, with Dunn’s test and Bonferroni correction applied for *post-hoc* analysis. Categorical variables were compared using the Chi-square test or Fisher’s exact test, as appropriate. A two-sided p-value of < 0.05 was considered statistically significant, with the Bonferroni-adjusted p-value used for interpreting *post-hoc* test results.

## Results

### To construct SIC and clinical characteristics of HBV-HCC based on immune exhaustion phenotype combined with immune protein profile

During the study period, a total of 80 HBV-HCC patients were tested for peripheral blood immune exhaustion phenotype and immune protein expression profiles. Among them, male patients accounted for 77.5%, with an average age of 60 years. 69 patients received TACE treatment, 2 patients underwent radical hepatectomy treatment, and 9 patients received conservative symptomatic treatment. The median follow-up overall survival (OS) time for the HBV-HCC patients was 39.6 months (**Table 1**).

**Table 1:** Clinical characteristics of HBV-HCC patients.

Variables	Overall (N = 80)
Gender	
Female	18 (22.5%)
Male	62 (77.5%)
Age	
Mean (SD)	60.0 (10.8)
Median [Min, Max]	61.0 [33.0, 86.0]
Tumor_Multiplicity	
Multiple	43 (53.8%)
Solitary	37 (46.3%)
Treatment_Methods	
Conservative	9 (11.3%)
Hepatectomy	2 (2.5%)
TACE	33 (41.3%)
TACE_MWA	5 (6.3%)
TACE_RFA	31 (38.8%)
Progression_Free_Survival_time	
Mean (SD)	23.1 (16.0)
Median [Min, Max]	22.6 [0.355, 44.0]
Overall_Survival_Time	
Mean (SD)	33.6 (14.1)
Median [Min, Max]	39.6 [0.0667, 45.9]

**Figure 2:**
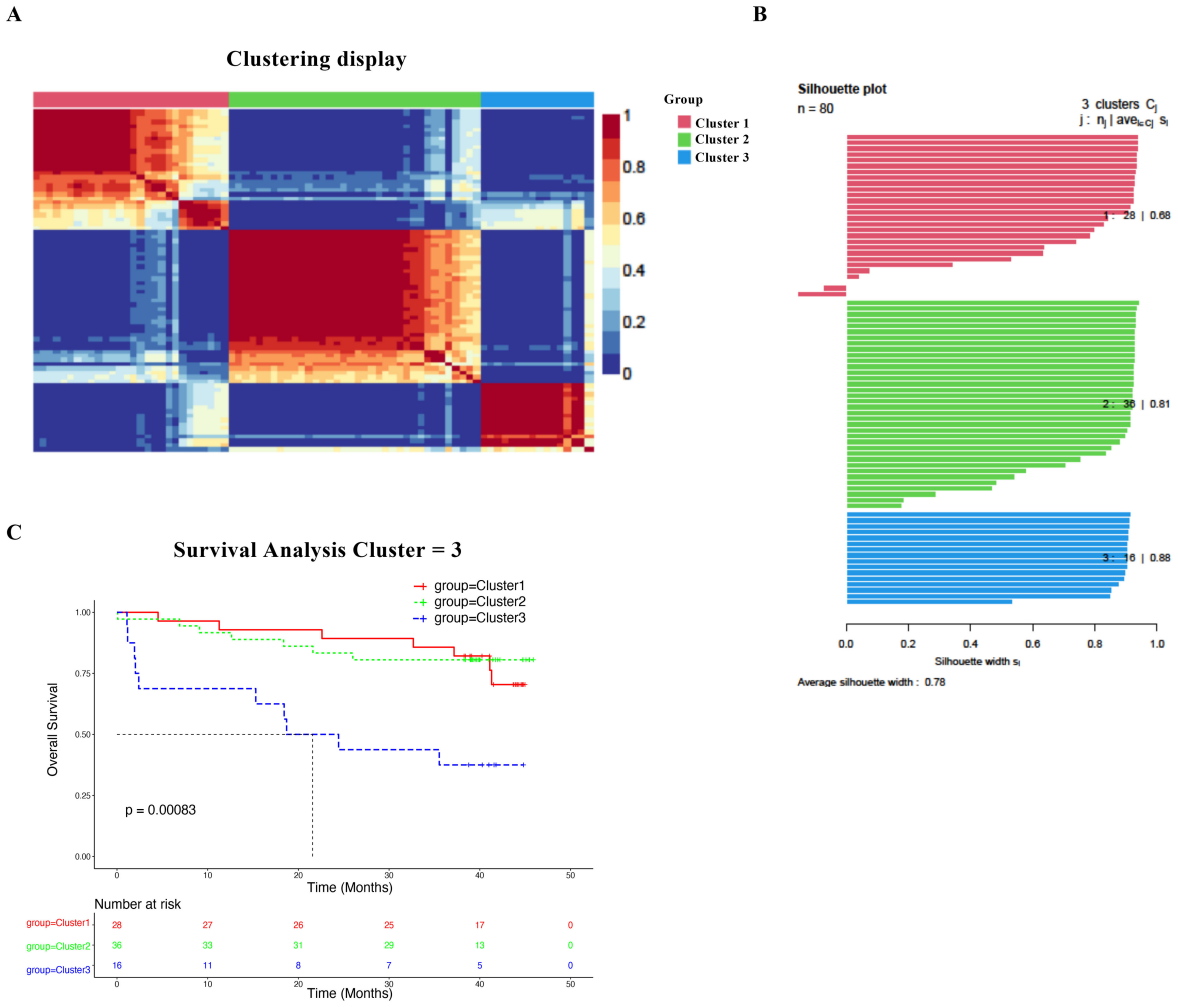
Construction of systemic immune classification (SIC) and clinical characteristics in HBV-HCC patients (N = 80). **(A)** The SIC cluster diagram is divided into three clusters. **(B)** Visualize clustering of silhouette plot by the silhouette coefficient. **(C)** Kaplan–Meier curve analysis shows different subtypes in the overall survival rate analysis of HBV-HCC patients. *P* values were obtained using the log-rank test.

We applied an unsupervised clustering algorithm to characterize the heterogeneity of HBV‐HCC patients through integrating two core dimensions of immune features: the immune exhaustion phenotype (encompassing 54 parameters) and immune protein expression profiles (encompassing 85 parameters). This analytical strategy stratified the patient cohort into three distinct subtypes, specifically Cluster 1 (n = 28), Cluster 2 (n = 36), and Cluster 3 (n = 16), which are visually presented in **Figure 2A**. The silhouette plot visualizes clustering by the silhouette coefficient (s), where s = (b − a)/max (a, b) (a: intra-cluster distance, b: nearest cluster distance; s ∈ [− 1, 1]). In the plot, 3 colors represent the subtype Cluster 1, Cluster 2, and Cluster 3. The length of each band reflects the s of the sample (longer = better clustering plausibility). Most bands exceed 0.5, indicating that the 3 subtypes of this cluster are optimal (**Figure 2B**). Moreover, we observed that patients in Cluster 1 exhibited superior OS compared to those in Clusters 2 and 3 (**Figure 2C**, p = 0.00083). Furthermore, based on the clinical characteristics of HBV-HCC patients, including liver function, liver protein synthesis function, coagulation function, inflammatory indicators, and tumor specificity, revealed Cluster 3 exhibited poorer liver function, decreased ability to contract proteins, and coagulation ability compared to Cluster 1 and Cluster 2, while inflammatory and tumor-specific indicators were significantly elevated (**Supplementary Figure S1**).

**Supplementary Table S1** demonstrates that the three immune subtypes (Cluster 1, Cluster 2, and Cluster 3) of HBV-HCC patients exhibit distinct clinical profiles, reflecting varying disease severity, liver function, tumor aggressiveness, and systemic inflammation. Cluster 3 stands out with significantly worse outcomes: higher rates of Child-Pugh B/C (87.5% vs. 21.4% [Cluster 1]/36.1% [Cluster 2]), indicating impaired liver reserve; more advanced tumors (81.2% BCLC C/D vs. 21.4% [Cluster 1]/30.5% [Cluster 2]; 68.8% tumors >5 cm vs. 28.6% [Cluster 1]/11.1% [Cluster 2]; 81.3% multiple tumors vs. 46.4% [Cluster 1]/47.2% [Cluster 2]); and elevated risks of complications (18.8% hepatic encephalopathy vs. 0% [Cluster 1]/2.8% [Cluster 2]; 75% portal hypertension vs. 25% [Cluster 1]/63.9% [Cluster 2]). Additionally, Cluster 3 showed worse liver function (median AST 61.25 vs. 25.25 [Cluster 1]/29.05 [Cluster 2] IU/L;median r-GGT 85.35 vs. 30.95 [Cluster 1]/30.35 [Cluster 2] IU/L), higher inflammatory markers (median CRP 31.6 vs. 4.0 [Cluster 1]/0.75 [Cluster 2] mg/L) and higher tumor profile (median AFP 402.95 vs. 5.5 [Cluster 1]/5.06 [Cluster 2]ng/ml). These differences highlight Cluster 3 as a high-risk subset requiring tailored management, while Clusters 1/2 display better treatment tolerance. In conclusion, the stratification underscores the clinical utility of immune subtyping in guiding personalized care for HBV-HCC.

### Characteristics of immune exhaustion in SIC of HBV-HCC patients

**Figure 3:**
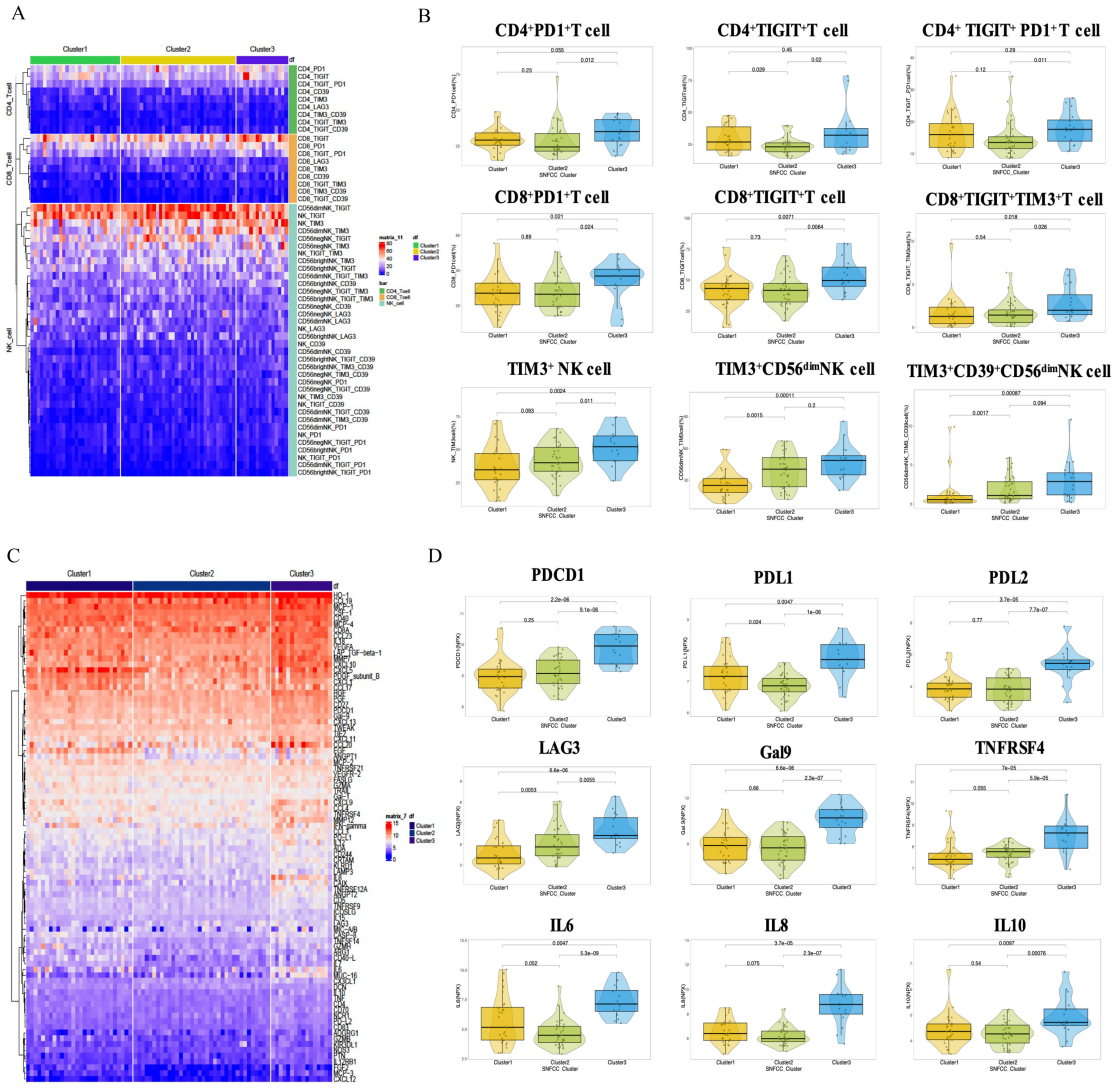
The immune cell exhaustion of HBV-HCC patients in systemic immune classification. **(A, B)** Comparison of immune exhaustion in SIC cluster diagram and significant difference box plots. **(C, D)** Comparison of immune proteins in SIC diagram and significant difference box plots.

In order to identify the phenotype of immune exhaustion in SIC, heat map clustering analysis was performed on various immune exhaustion phenotypes in different subsets of CD8^+^T cells, CD4^+^T cells and NK cells. The circle-gate strategy for T cells and NK cells in multicolor flow cytometry is shown in **Supplementary Figure S2**. Heat maps show the expression of immune exhaustion phenotypes across different SIC (**Figure 3A**). The Cluster 3 exhibited significantly higher expression levels of exhaustion markers in CD8^+^PD-1^+^ T cells, CD8^+^TIGIT^+^ T cells, CD8^+^TIGIT^+^TIM-3^+^ T cells, TIM-3^+^ NK cells, and TIM-3^+^CD39^++^CD56_dim_ NK cells compared to Cluster 1 and 2 (**Figure 3B**, **Supplementary Figure S3**, p < 0.05). The expression profiles of 92 immune-oncology proteins expression profiles were analyzed, and Cluster 3 showed significantly higher expression of inhibitory molecules (PDCD1, PDL1, PDL2, LAG3, CD244), inflammatory factors (IL6, IL8, IL10, IL12, IL15, IL18), and chemokines (CCL3, CCL4, CCL17, CCL19, CCL20) than Cluster 1/2 (**Figures 3C, D**, **Supplementary Figure S4**, p < 0.05).

### Prediction of clinical response in HBV-HCC patients after TACE treatment

**Figure 4:**
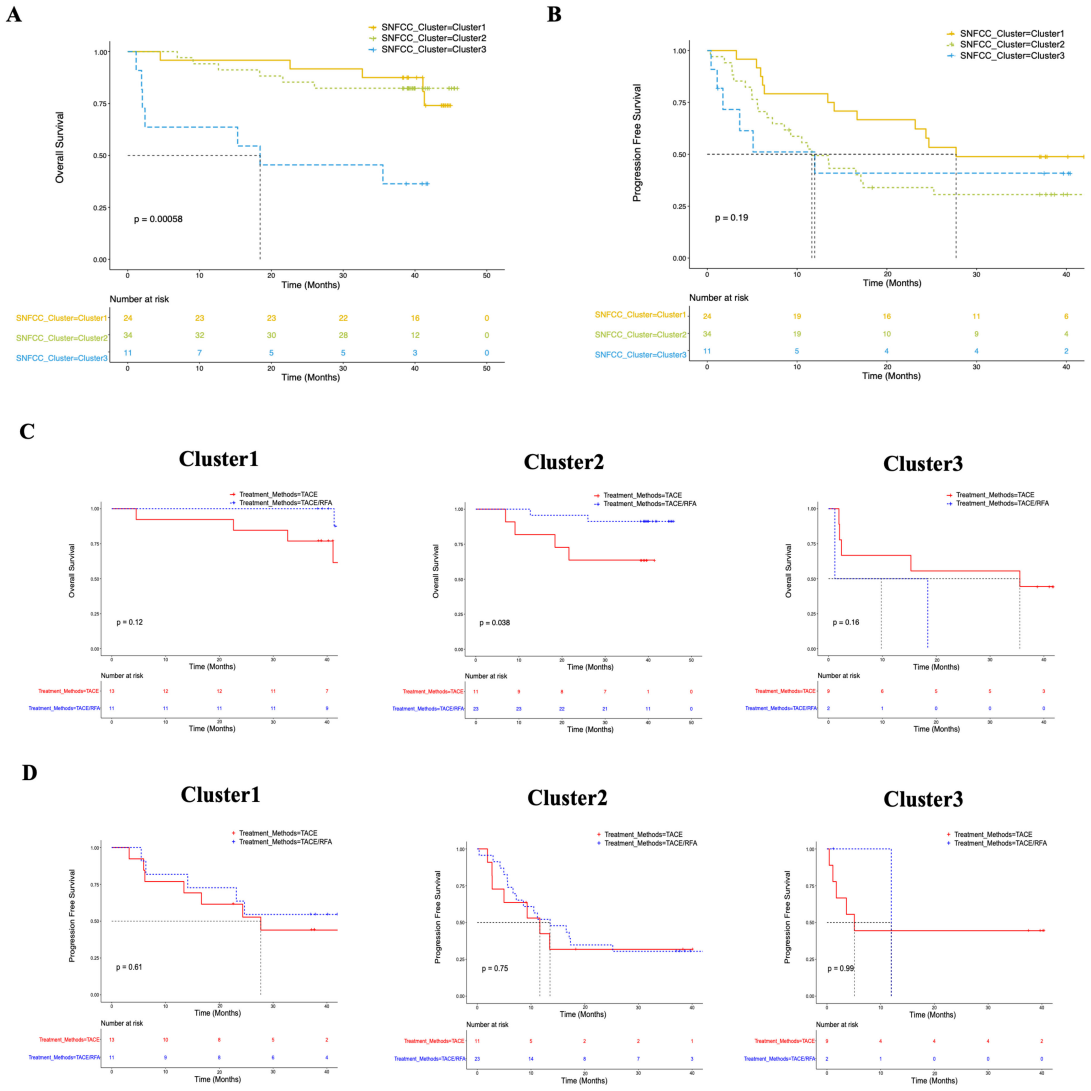
Survival analysis after TACE treatment in HBV-HCC patients (N = 69) with different systemic immune classification. **(A)** Kaplan-Meier curve analysis shows that different immune subtypes can predict the overall survival rate of HBV-HCC patients. **(B)** Kaplan-Meier curve analysis shows that different immune subtypes can predict the progression-free survival rate of HBV-HCC patients. **(C)** Kaplan-Meier curve analysis shows that TACE alone and TACE combination therapy can predict the overall survival rate of HBV-HCC patients. **(D)** Kaplan-Meier curve analysis shows that TACE alone and TACE combination therapy can predict the progression-free survival rate of HBV-HCC patients. *P* values were obtained using the log-rank test.

The study included a real-world study of 80 HBV-HCC patients, of which 69 received TACE treatment, including TACE alone (N = 33) and TACE combined with RFA/MWA (N = 36). Assessing the effects of different SIC on clinical responses in patients with HBV-HCC after TACE treatment, the overall survival rate of patients in Cluster 3 was significantly lower than that in Cluster 1 and Cluster 2 (**Figure 4A**, p = 0.00058). However, the difference in progression-free survival was not statistically significant (**Figure 4B**, p = 0.19). A further comparison of the overall survival effects of TACE alone versus TACE combined with RFA/MWA treatment across different immune subtypes revealed that the overall survival rate for patients receiving the combined treatment was superior to that of those treated with TACE alone in Cluster 1 and Cluster 2. However, no advantage of the combined treatment was observed in the Cluster 3 (**Figure 4C**). While the small sample size in this subgroup is a limitation, we posit that the primary reason is biological: the profoundly exhausted and immunosuppressive microenvironment in Cluster 3 is inherently resistant to immune-mediated synergy between locoregional therapies. Therefore, we suggest that for Cluster 1 and Cluster 2, topical treatment should be strengthened, while for Cluster 3, a shift to systemic immunomodulation should be made. This study provides ideas on the most effective individualized combination therapy for HCC patients receiving TACE using immune phenotypes. Additionally, the analysis of progression-free survival among patients treated with TACE across the three immune subtypes did not demonstrate any significant differences (**Figure 4D**).

### Screen the core indicators influencing the clinical response after TACE treatment

**Figure 5:**
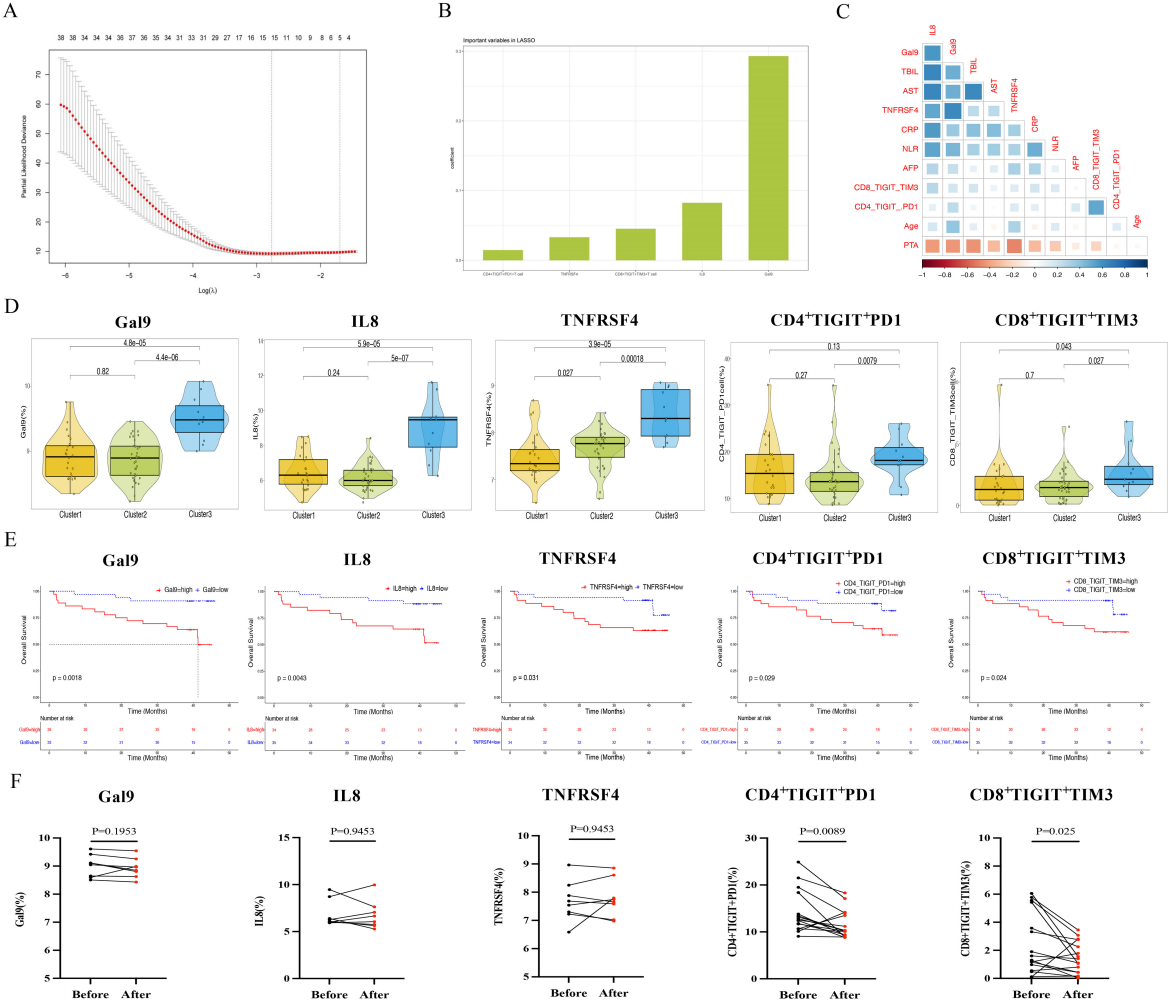
Establishing core indicators related to overall survival of HBV-HCC patients after TACE treatment. **(A, B)** LASSO regression screening for prognostic indicators and importance ranking after TACE treatment. **(C)** Correlation analysis between core indicators and clinical features. **(D)** Box plot comparison of core indicators and SIC. **(E)** Kaplan-Meier curve analysis of the high and low core indicators groups. **(F)** Comparison of core indicators before and after TACE treatment using paired t-test.

Subsequently, the core indicators of overall survival after TACE treatment were clearly focused, and the indicators of differences between immune exhaustion phenotype and immune protein were screened by LASSO regression (**Figure 5A**). Finally, five core indicators were obtained to better evaluate the impact of immune classification and prognosis. Among them, the five core indicators include 3 immune proteins (Gal9, IL8, TNFRSF4) and 2 immune exhaustion phenotypes (CD4^+^TIGIT^+^PD1^+^T cells, CD8^+^TIGIT^+^TIM3^+^T cells), with the Gal9 protein being the most important in LASSO (**Figure 5B**, **Supplementary Figure S5**). To complement our peripheral blood findings, we assessed Gal9 expression in the tumor microenvironment. Immunohistochemical staining showed significantly higher Gal9 expression in tumor tissues (**Supplementary Figure S6A**) than in adjacent tissues (**Supplementary Figure S6B**; p < 0.0001). Meanwhile, there was a significant positive correlation between Gal9 levels and the immune exhaustion phenotype of CD4^+^TIGIT^+^PD1^+^T cells (**Supplementary Figure S6C**, p = 0.0111, r = 0.3039). The correlation between the five core indicators and clinical features showed that Gal9 and IL8 were significantly positively correlated with TBIL, AST, CRP, NLR, and negatively correlated with PTA (**Figure 5C**). The expression levels of five core indicators of Cluster 3 were higher than those of Cluster 1 and Cluster 2 (**Figure 5D**, p < 0.05). After analyzing the clinical response of the five core indicators and patients after TACE treatment, it was found that the group with high expression of core indicators had a lower overall survival rate (**Figure 5E**, p < 0.05).

Further examination of the five core indicators after TACE treatment for 6 months compared to baseline levels showed that although the levels of Gal9, IL8, and TNFRSF4 immune proteins did not change before and after treatment, the expression levels of CD4^+^TIGIT^+^PD1^+^T cells and CD8^+^TIGIT^+^TIM3^+^T cells in the immune exhaustion phenotype were significantly decreased after TACE treatment compared to baseline levels (**Figure 5F**).

### Establish a riskScore for clinical response after TACE treatment

**Figure 6:**
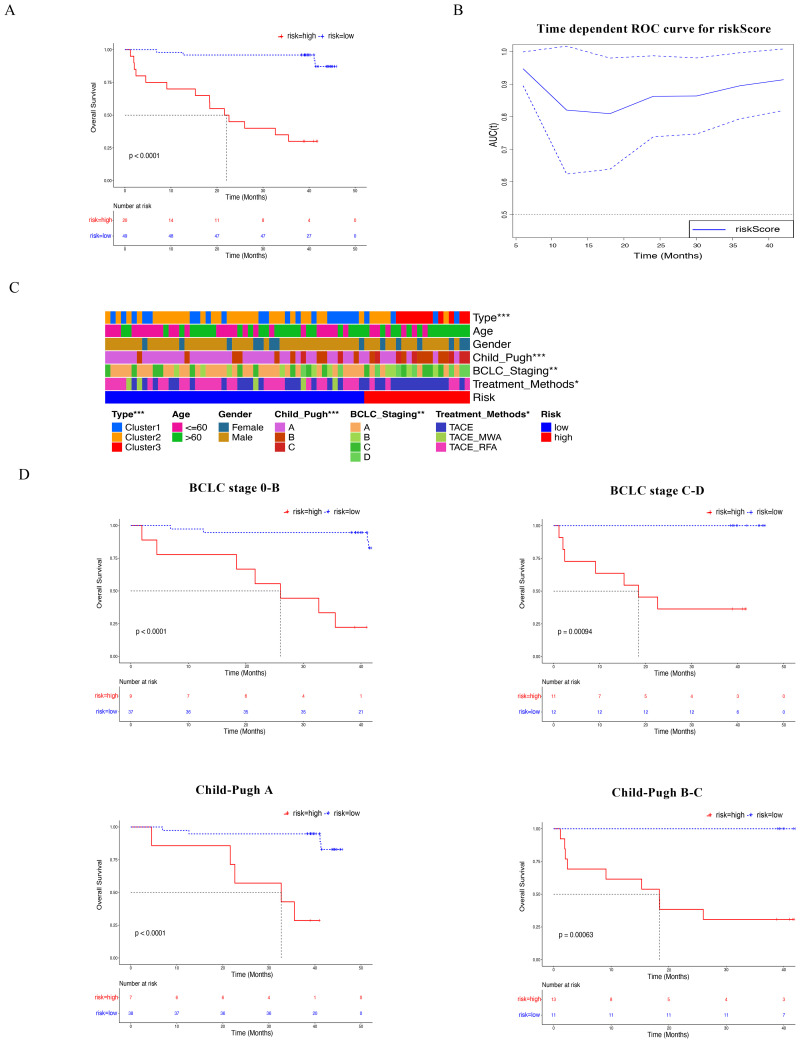
LASSO regression is used to construct a risk scoring system to predict TACE treatment response. **(A)** The analysis and comparison of Kaplan-Meier curve analysis predict the response of HBV-HCC patients after TACE treatment based on high and low risk scores. **(B)** RiskScore predicts the ROC curve of HBV-HCC patients after TACE treatment. **(C)** Cluster analysis of risk scores and different clinical characteristics of HBV-HCC patients. **(D)** Kaplan-Meier curve analysis was used to analyze the survival rate of HBV-HCC patients in different BCLC stages and Child-Pugh grades.

In order to better evaluate the efficacy of TACE treatment for HBV-HCC, a focused riskScore was constructed based on five core indicators, with the riskScore formula= 0.082411012 * IL8 + 0.293025556 * Gal9 + 0.033152133 * TNFRSF4 + 0.014714986 * CD4^+^TIGIT^+^PD1^+^T cells + 0.045277334 * CD8^+^TIGIT^+^TIM3^+^T cells. **Supplementary Figure S7A** shows the distribution characteristics and groupings of risk scores in the study cohort. The blue dashed line in the plot indicates the cut-off value of 3.87, which divides the sample into low-risk (left, risk = low) and high-risk (right, risk = high) groups. Below this critical value, there is no significant overlap between the scoring intervals of the high-risk and low-risk groups, thereby ensuring the clarity of the grouping. **Supplementary Figure S7B** illustrates the overall survival distribution of patients. It is evident that the overall survival time of the high-risk group is significantly shorter than that of the low-risk group, suggesting that this critical value effectively distinguishes patients with differing prognoses. Based on the distribution characteristics of the risk score and its prognostic value for survival outcomes, the value of 3.87 was ultimately determined as the critical threshold for the risk score in this study, facilitating the distinction between high and low-risk groups in terms of both biological and clinical significance. Survival analysis found that the overall survival time of patients in the high-risk group was significantly lower than that of patients in the low-risk group (**Figure 6A**, p < 0.0001). Meanwhile, the riskScore system predicts that the time-dependent AUC values of HBV-HCC patients after TACE treatment are all above 0.8 (**Figure 6B**). By combining this riskScore system with major clinical indicators such as age, AFP, NLR, and CRP, the Nomogram model was constructed. The AUC values of the ROC curves of the Nomogram model at 1 year, 2 years, and 3 years were 0.885, 0.927, and 0.946, respectively, which were higher than the AUC values of riskScore at different times (**Supplementary Figures S8A, B**).

Next, we fully demonstrated the role of this riskScore system in different clinical subgroup of HBV-HCC patients. The high-risk group of patients is mainly distributed in Child-Pugh B and C-grade, BCLC staging C-D, underlying coronary heart disease, comorbidities with hepatic encephalopathy and vascular metastasis, larger tumor diameter, multiple tumors, poorer liver function, higher inflammatory indicators NLR and CRP, and higher AFP (**Figure 6C**, **Supplementary Table S2**, p<0.05). Furthermore, for patients with BCLC stages A-B and C-D, or patients with different Child-Pugh grades, the overall survival rate of the high-risk group was lower (**Figure 6D**). Further, in the subgroups of TACE alone and TACE combined with RFA/MWA, the overall survival rate of patients in the high-risk group was significantly lower than that in the low-risk group (**Supplementary Figure S9**). In order to further verify the value of riskScores system in different clinical substrates, we analyzed in BCLC stage 0-B, BCLC stage C-D, Child-Pugh A, and Child-Pugh B-C respectively. The AUC values obtained from these analyses were all greater than 0.8 (**Supplementary Figure S10**). Therefore, the above results support the clinical application value of establishing a predictive efficacy evaluation riskScore system for HBV-HCC after TACE treatment based on SIC.

## Discussion

In this study, we developed and validated a novel Systematic Immune Classification (SIC) based on the non-invasive peripheral blood immune exhaustion phenotype of patients with HBV-related hepatocellular carcinoma (HBV-HCC). The optimal silhouette coefficient confirmed the robustness of our three-cluster solution (Cluster 1, Cluster 2 and Cluster 3), which demonstrated significant differences in overall survival (OS) among these subtypes. This SIC framework addresses a critical limitation in current HCC management, where prognostic models predominantly rely on clinical and radiological parameters (11–15). Few studies have employed the non-invasive peripheral blood immune exhaustion phenotype to anticipate clinical responses. Given the considerable heterogeneity in patients’ immune profiles, there is a pressing need for immune-related biomarkers to enhance prognostic assessment. Unlike tissue-based immune classifications that require invasive biopsies and fail to capture SIC, our peripheral blood-based approach provides a practical tool for serial immune monitoring throughout transarterial chemoembolization (TACE) treatment. The distinct survival outcomes across clusters validate the clinical relevance of SIC in stratifying HBV-HCC patients undergoing TACE.

Cluster 3 exhibited an immunosuppressive/exhausted phenotype, which was associated with significantly poorer OS post-TACE. Patients in this cluster also demonstrated compromised liver and coagulation function alongside increased tumor burden. This aggressive phenotype is likely driven by HBV-HCC-specific molecular mechanisms. HBV-related factors (e.g., viral proteins, genomic integration events) may directly promote tumorigenesis and shape an immunosuppressive microenvironment, potentially explaining the adverse outcomes in Cluster 3 (22). Notably, Cluster 3 was distinguished by concurrent elevation of soluble mediators (Gal9, IL8) and enrichment of immune exhaustion phenotypes (CD4^+^TIGIT^+^PD-1^+^ and CD8^+^TIGIT^+^TIM-3^+^). Building on these findings, we propose two mechanistic hypotheses: (1) a Gal9–TIM3 positive feedback loop driving and sustaining T cell exhaustion, and (2) an IL8–myeloid-derived suppressor cell (MDSC) axis recruiting immunosuppressive cells and inducing T cell dysfunction. These hypotheses distinguish our study from prior associative reports by directly linking specific soluble factors to a co-inhibitory receptor-defined exhaustion signature in a clinically relevant patient subgroup (23, 24). To validate these mechanisms, we will employ recombinant proteins and pathway inhibitors *in vitro*, alongside an intra-nodal immunocompetent mouse model. These experiments aim to establish causality and evaluate the therapeutic potential of targeting these pathways, addressing the critical unmet need to overcome TACE resistance in this high-risk population.

In this study, the expression level of Gal9 was revealed by the analysis of peripheral blood and tumor tissue. Gal9, a ligand for TIM3, is highly expressed in subsets of advanced HCC cells, indicating a negative prognosis (25). The Gal9/TIM3 signaling pathway is known to inhibit the immune functions of T cells and NK cells (26), suggesting that targeting Gal9 may represent an effective and promising immunotherapy strategy for HCC. Research has indicated that the mechanism behind the increased expression of Gal9 involves miR-9 3-5p targeting an epigenetic regulator, which leads to immune escape. Clinically, HCC is closely associated with elevated Gal9 levels and resistance to anti-PD1 treatment (27). Combined with existing studies, we speculate that it may play a complex and critical role in immune regulation after TACE treatment. After TACE treatment, the high expression of Gal9 may suppress the function of T cells and NK cells and weaken the efficacy of the anti-tumor immune response. The formation of this immunosuppressive state may be a potential mechanism for immune tolerance and tumor recurrence after TACE.

The clinical utility of our SIC was demonstrated through its strong association with TACE treatment outcomes. Patients in Cluster 3 showed significantly poorer overall survival and progression-free survival following TACE, indicating their resistance to conventional locoregional therapy (28). Importantly, our analysis revealed that while TACE combination therapy with RFA/MWA benefited patients in Clusters 1 and 2, it provided no additional survival advantage for those in Cluster 3. This finding underscores the importance of SIC-guided treatment stratification: for immune-competent phenotypes (Clusters 1 and 2), intensification of local therapy remains appropriate; however, for the immune-exhausted phenotype (Cluster 3), alternative strategies that combine TACE with systemic immunomodulation may be necessary to address the inherent treatment resistance (29–31).

To enhance clinical applicability, we developed a quantitative riskScore that incorporates five core indicators: three immune proteins (Gal9, IL8, TNFRSF4) and two immune exhaustion phenotypes (CD4^+^TIGIT^+^PD-1^+^ T cells and CD8^+^TIGIT^+^TIM-3^+^ T cells). This riskScore demonstrated superior predictive performance (AUC >0.8) for overall survival compared to conventional clinical models. The strength of the model lies in its integration of both immune exhaustion phenotype and immune protein, providing a more comprehensive assessment of the SIC than single-platform assays (18, 19, 32).

Our findings position the SIC and the derived riskScore as valuable tools for personalizing HCC management. For patients in Cluster 3, who exhibit profound immune exhaustion characterized by the expression of multiple co-inhibitory receptors, single-agent immunotherapy is likely insufficient (33–35). Instead, our data provide a strong rationale for exploring TACE in combination with dual or triple checkpoint blockade targeting PD-1 alongside TIGIT and/or TIM3 (26, 36, 37). Furthermore, targeting upstream mediators such as the IL8/CXCR2 axis to inhibit MDSC recruitment or neutralizing Gal9 to relieve TIM3-mediated suppression represents promising strategies to reverse immunosuppression in this high-risk population (27, 38).

Several limitations of this study should be noted. Firstly, this investigation represents a preliminary, small-sample analysis of non-invasive immune profiling within a real-world cohort of HBV-HCC patients undergoing TACE; thus, future validation in larger, prospective studies is essential. Secondly, the limited availability of samples hindered longitudinal tracking of immune classification and the mechanistic dissection of TACE-induced immunomodulation. Thirdly, the predictive value of our immune classification for subsequent immunotherapy following TACE remains to be assessed, which is a focal point of our ongoing research. Finally, as highlighted in recent commentaries, HBV continues to be a significant oncogenic driver, and the intricate pathogenesis of HBV-HCC requires sustained investigative efforts (39). While our work represents progress through non-invasive immune monitoring, it also underscores the ongoing challenges within this field.

## Conclusions

In summary, we established a non-invasive ​​SIC for HBV-HCC patients after TACE treatment, which can predict the clinical response of patients and indicate the immune exhaustion phenotypes (CD4^+^TIGIT^+^PD1^+^T cells, CD8^+^TIGIT^+^TIM3^+^T cells) and plasma proteins (Gal9, IL8, TNFRSF4) were closely correlated with overall survival after TACE treatment. Future investigations involving larger external cohorts will be necessary to clarify the potential applications of immune classification in the management of HBV-HCC.

## Data Availability

The original contributions presented in the study are included in the article/**Supplementary Material.** Further inquiries can be directed to the corresponding authors.
