# Supplementary material for: Systemic immune classification related to immune exhaustion evaluates the clinical response of patients with HBV-HCC after transarterial chemoembolization
Source: Front Immunol. 2025 Dec 19;16:1629052. doi: 10.3389/fimmu.2025.1629052 (PMC12757348; doi:10.3389/fimmu.2025.1629052)
Supplement: Supplementary file 1 [file DataSheet1.docx]

**Table**

TableS1.Clinical characteristics of HBV-HCC patients with different systematic immune classification.

| **Variables** | **Cluster 1** | **Cluster 2** | **Cluster 3** | **P value** |
| --- | --- | --- | --- | --- |
|  | **（n=28）** | **(n=36)** | **(n=16)** |  |
| **Demographic characteristics (%)** | |  |  |  |
| Age (mean±SD) | 60.04±9.70 | 57.61±10.17 | 64.06±11.96 | 0.164 |
| Gender (Female/Male) | 4/24(14.3%/85.7%) | 7/29(19.4%/80.6%) | 7/9(43.8%/56.3%) | <0.0001 |
| Chlid_Pugh (A/B/C) | 22/4/2(78.6%/14.3%/7.1%) | 23/10/3(63.9%/27.8%/8.3%) | 2/8/6(12.5%/50.0%/37.5%) | <0.0001 |
| BCLC_Staging (A/B/C/D) | 16/6/4/2(57.1%/21.4%/14.3%/7.1%） | 20/5/8/3(55.6%/13.9%/22.2%/8.3%) | 1/2/7/6(6.3%/12.5%/43.8%/37.5%) | <0.0001 |
| Hypertension (Yes/No) | 11/17(39.3%/60.7%) | 10/26(27.8%/72.2%) | 6/10(37.5%/62.5%) | 0.589 |
| Diabetes (Yes/No) | 7/21(25%/75%) | 10/26(27.8%/72.2%) | 4/12(25%/75%) | 0.961 |
| Coronary_Disease (Yes/No) | 1/27(3.6%/96.4%) | 2/34(5.6%/94.4%) | 0/16(0%/100%) | 0.622 |
| Upper_Gastrointestinal_Hemorrhage (Yes/No) | 3/25(10%/7%/89.3%) | 0/36(0%/100%) | 2/14(12.5%/87.5%) | 0.110 |
| Hepatic_Encephalopathy (Yes/No) | 0/28(0%/100%) | 1/35(2.8%/97.2%） | 3/13(18.8%/81.2%) | 0.016 |
| Vascular_Metastasis (Yes/No) | 2/26(7.1%92.9%) | 2/34(5.6%/94.4%) | 4/12(25%/75%) | 0.080 |
| Distant_Metastasis (Yes/No) | 4/24(14.3%/85.7%) | 9/27(25%/75%) | 9/7(56.2%/43.8%) | 0.010 |
| Portal_Hypertension (Yes/No) | 7/21(25%/75%) | 23/13(63.9%/36.1%) | 12/4(75%/25%) | 0.001 |
| Tumor_Size (≤5cm/>5cm) | 20/8(71.4%/28.6%) | 32/4(88.9%/11.1%) | 5/11(31.2%/68.8%) | <0.0001 |
| Tumor_Multiplicity (Solitary/Multiple) | 15/13(53.6%/46.4%) | 19/17(52.8%/47.2%) | 3/13(18.7%/81.3%) | <0.0001 |
| Treatment (Conservative/  Minimally_Invasive/Resection) | 3/24/1(10.7%/85.7%/3.6%) | 1/34/1(2.8%/94.4%/2.8%) | 5/11/0(31.3%/68.8%/0%) | <0.0001 |
| Treatment_Methods (Conservative/Hepatectomy/TACE/TACE_MWA/TACE_RFA) | 3/1/13/1/10(10.7%/3.6%/46.4%/3.6%/35.7%) | 1/1/11/4/19(2.8%/2.8%/30.6%/11.1%/52.8%) | 5/0/9/0/2(31.3%/0%/56.3%/0%/12.5%) | <0.0001 |
| **Laboratory parameters** |  |  |  |  |
| WBC (10^9^/L) | 5.13(4.33,6.64) | 3.62(2.65,4.83) | 5.46(3.19,7.87) | 0.001 |
| Neutrophil_Count (10^9^/L) | 3.34(2.30,4.87) | 1.76(1.46,3.18) | 3.40(1.91,6.55) | 0.001 |
| Lymphocyte_Count (10^9^/L) | 1.11(0.84,1.69) | 0.88(0.53,1.39) | 0.79(0.45,1.26) | 0.150 |
| N/L ratio | 2.97(2.18,5.45) | 2.01(1.46,3.68) | 4.11(2.43,9.11) | 0.014 |
| RBC (10^9^/L) | 4.45±0.85 | 3.98±0.68 | 3.34±0.81 | <0.0001 |
| HGB (g/L) | 136.38±25.78 | 124.22±23.83 | 105.21±24.70 | 0.001 |
| PLT (10^9^/L) | 118.00(91.4,190.38) | 76.5(48.63,93.85) | 103.55(78.25,172.5) | <0.0001 |
| BUN (μmol/L) | 5.12(4.39,6.07) | 4.60(3.95,5.81) | 6.76(3.81,8.29) | 0.193 |
| CR (μmol/L) | 68.35(57.05,77.25) | 61.8(52.35,69.5) | 64.45(61.03,85.25) | 0.191 |
| ALT (IU/L) | 20.25(16.13,39.13) | 21.35(17.3,31.58) | 43.15(19.28,69.88) | 0.081 |
| AST (IU/L) | 25.25(20.83,51.03) | 29.05(21.8,38.03) | 61.25(47.00,115.68) | <0.0001 |
| TBIL (μmol/L) | 16.60(11.00,21.75) | 19.95(12.48,26.53) | 31.5(18.43,65.53) | 0.005 |
| DBIL (μmol/L) | 6.85(4.08,10.08) | 8.2(5.45,11.73) | 19.45(9.05,50.58) | 0.001 |
| ALB (g/L) | 39.00(33.90,41.90) | 36.85(33.05,40.28) | 29.5(27.85,32.45) | <0.0001 |
| r-GGT (IU/L) | 30.95(20.93,72.50) | 30.35(20.95,53.75) | 85.35(37.00,258.45) | 0.003 |
| PTA (%) | 86.89±19.36 | 80.83±19.95 | 69.94±15.65 | 0.001 |
| AFP (ng/ml) | 5.5(1.9,165.3) | 5.06(2.4,61.68) | 402.95(10.45,2000) | 0.022 |
| CRP (mg/L) | 4.00(0.65,16.95) | 0.75(0.30,1.85) | 31.6(7.28,66.65) | <0.0001 |
| **Hepatitis_B_Related_Characteristics (%)** | |  |  |  |
| HBV-DNA  (Not_Detected/<20IU/ml/>20IU/ml) | 11/5/12(39.3%/17.9%/42.9%) | 14/9/13(38.9%/25.0%/36.1%) | 8/1/7(50%/6.3%/43.8%) | 0.606 |
| HBeAg(<1 S/CO/>1 S/CO) | 18/10(64.3%/35.7%) | 29/7(80.6%/19.4%) | 12/4(75%/25%) | 0.338 |
| HBeAb(<1 S/CO/>1 S/CO) | 14/14(50%/50%) | 20/16(55.6%/44.4%) | 7/9(43.8%/56.3%) | 0.724 |
| HBcAb(<1S/CO/>1 S/CO/Not_Applicable) | 0/18/10(0%/64.3%/35.7%) | 2/32/2(5.6%/88.9%/5.6%) | 1/10/5(6.3%/62.5%/31.2%) | 0.027 |

Abbreviations: HBV, hepatitis B virus; BCLC, Barcelona Clinic Liver Cancer classification system; WBC, white blood cell; N/L, neutrophil-lymphocyte ratio; RBC, red blood cell; HGB, haemoglobin; PLT, platelet; BUN, Blood Urea Nitrogen; CR, creatinine; ALT, alanine aminotransferase; AST, aspartate aminotransferase; TBIL, total bilirubin; DBIL, Direct Bilirubin; ALB, albumin; γ-GGT, γ-glutamyl transferase; PTA, prothrombin time activity; AFP, alpha-fetoprotein; CRP, C-Reactive Protein.

TableS2. Clinical characteristics of HBV-HCC patients after TACE treatment in high and low risk groups

| **Variables** | **High Risk** | **Low Risk** | **P value** |
| --- | --- | --- | --- |
|  | **（n=20）** | **(n=49)** |  |
| **Demographic characteristics (%)** |  |  |  |
| Age (mean±SD) | 61.80±10.30 | 58.08±10.21 | 0.176 |
| Gender (Female/Male) | 6/14(30%/70%) | 8/41(16.3%/83.7%) | 0.200 |
| Hypertension (Yes/No) | 6/14(30%/70%) | 15/34(30.6%/69.4%) | 0.960 |
| Diabetes (Yes/No) | 4/16(20%/80%) | 15/34(30.6%/69.4%) | 0.371 |
| Coronary_Disease (Yes/No) | 2/18(10%/90%) | 0/49(0%/100%) | 0.025 |
| Upper_Gastrointestinal_Hemorrhage (Yes/No) | 2/18(10%/90%) | 3/46(6.1%/93.9%) | 0.573 |
| Hepatic_Encephalopathy (Yes/No) | 2/18(10%/90%) | 0/49(0%/100%) | 0.025 |
| Vascular_Metastasis (Yes/No) | 5/15(25%75%) | 1/48(2%/98%) | 0.002 |
| Distant_Metastasis (Yes/No) | 7/13(35%/65%) | 10/39(20.4%/79.6%) | 0.202 |
| Portal_Hypertension (Yes/No) | 13/7(65%/35%) | 22/27(44.9%/55.1%) | 0.130 |
| Tumor_Size (≤5cm/>5cm) | 8/12(40%/60%) | 43/6(87.8%/12.2%) | <0.0001 |
| Tumor_Multiplicity (Solitary/Multiple) | 6/14(30%/70%) | 28/21(57.1%/42.9%) | 0.041 |
| **Laboratory parameters** |  |  |  |
| WBC (10^9^/L) | 5.97±3.32 | 4.35±1.74 | 0.050 |
| Neutrophil_Count (10^9^/L) | 4.25±3.00 | 2.69±1.34 | 0.035 |
| Lymphocyte_Count (10^9^/L) | 0.94±0.46 | 1.16±0.62 | 0.151 |
| N/L ratio | 3.45(2.2,7.91) | 2.33(1.54,3.56) | 0.160 |
| RBC (10^9^/L) | 3.63±0.87 | 4.28±0.77 | 0.003 |
| HGB (g/L) | 114.01±26.68 | 132.06±25.11 | 0.010 |
| PLT (10^9^/L) | 138.52±99.84 | 101.72±52.68 | 0.132 |
| BUN (μmol/L) | 5.41±2.37 | 5.00±1.46 | 0.471 |
| CR (μmol/L) | 64.23±11.75 | 67.49±16.15 | 0.416 |
| ALT (IU/L) | 49.66±40.02 | 28.19±28.78 | 0.038 |
| AST (IU/L) | 56.75(31,114.18) | 28.2(21.3,37.75） | 0.001 |
| TBIL (μmol/L) | 25.5(18.78,46.15) | 16.5(11.05,23.1) | <0.0001 |
| DBIL (μmol/L) | 14.35(8.83,30.05) | 7.0(4.15,9.05) | <0.0001 |
| ALB(g/L) | 32.26±4.93 | 37.43±5.37 | <0.0001 |
| r-GGT (IU/L) | 83.75(24.7,281.9) | 29.9(21,53.3) | 0.008 |
| PTA (%) | 69.5±19.78 | 86.51±17.98 | 0.001 |
| AFP (ng/ml) | 65.95(3.33,1350.21) | 4.6(2.05,64.45) | 0.015 |
| CRP (mg/L) | 16.80(3.4,64.98) | 0.7(0.3,3.05) | <0.0001 |
| **Hepatitis_B_Related_Characteristics (%)** | |  |  |
| HBV-DNA  (Not_Detected/<20IU/ml/>20IU/ml) | 10/1/9(50%/5%/45%) | 20/11/18(40.8%/22.4%/36.7%) | 0.222 |
| HBeAg(<1 S/CO/>1 S/CO) | 18/2(90%/10%) | 33/16(67.3%/32.7%) | 0.052 |
| HBeAb(<1 S/CO/>1 S/CO) | 12/8(60%/40%) | 23/26(46.9%/53.1%) | 0.325 |
| HBcAb(<1S/CO/>1 S/CO/Not_Applicable) | 2/14/4(10%/70%/20%) | 1/37/11(2%/75.5%/22.4%) | 0.338 |

Abbreviations: HBV, hepatitis B virus; BCLC, Barcelona Clinic Liver Cancer classification system; WBC, white blood cell; N/L, neutrophil-lymphocyte ratio; RBC, red blood cell; HGB, haemoglobin; PLT, platelet; BUN, Blood Urea Nitrogen; CR, creatinine; ALT, alanine aminotransferase; AST, aspartate aminotransferase; TBIL, total bilirubin; DBIL, Direct Bilirubin; ALB, albumin; γ-GGT, γ-glutamyl transferase; PTA, prothrombin time activity; AFP, alpha-fetoprotein; CRP, C-Reactive Protein.


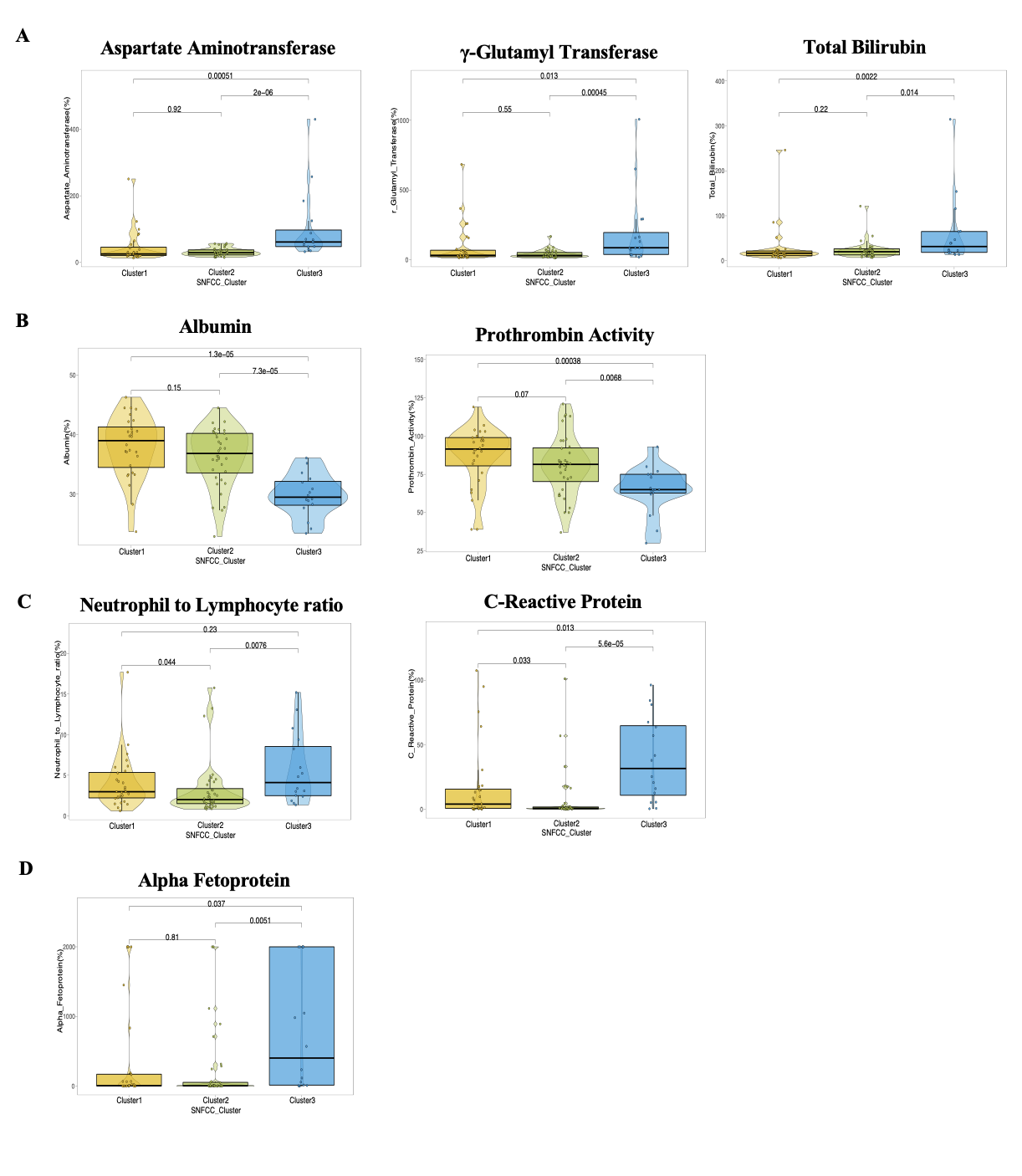


FigureS1. Comparison of expression levels of liver function, coagulation function, inflammatory markers, and tumor markers in HBV-HCC patients with different systemic immune classification.


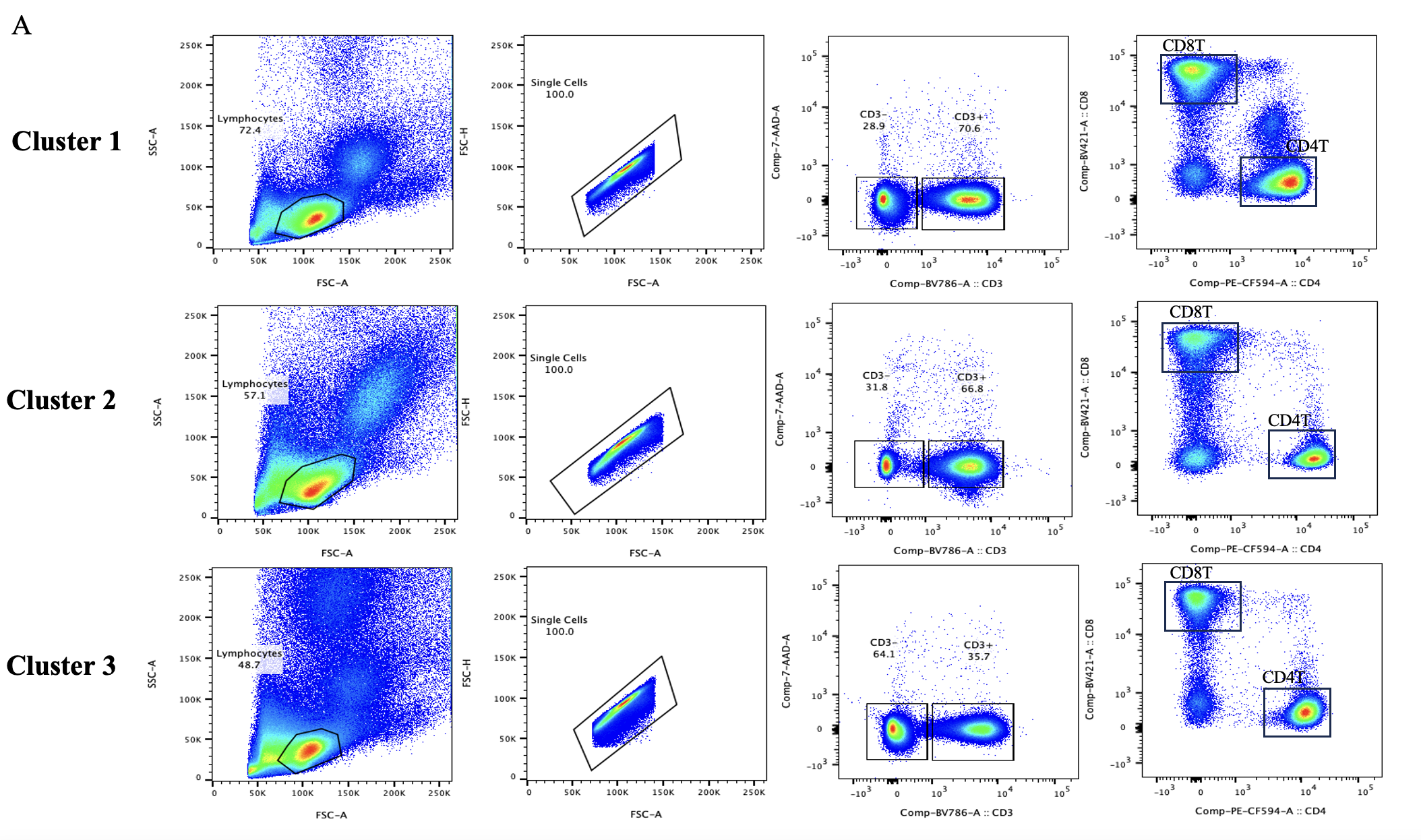


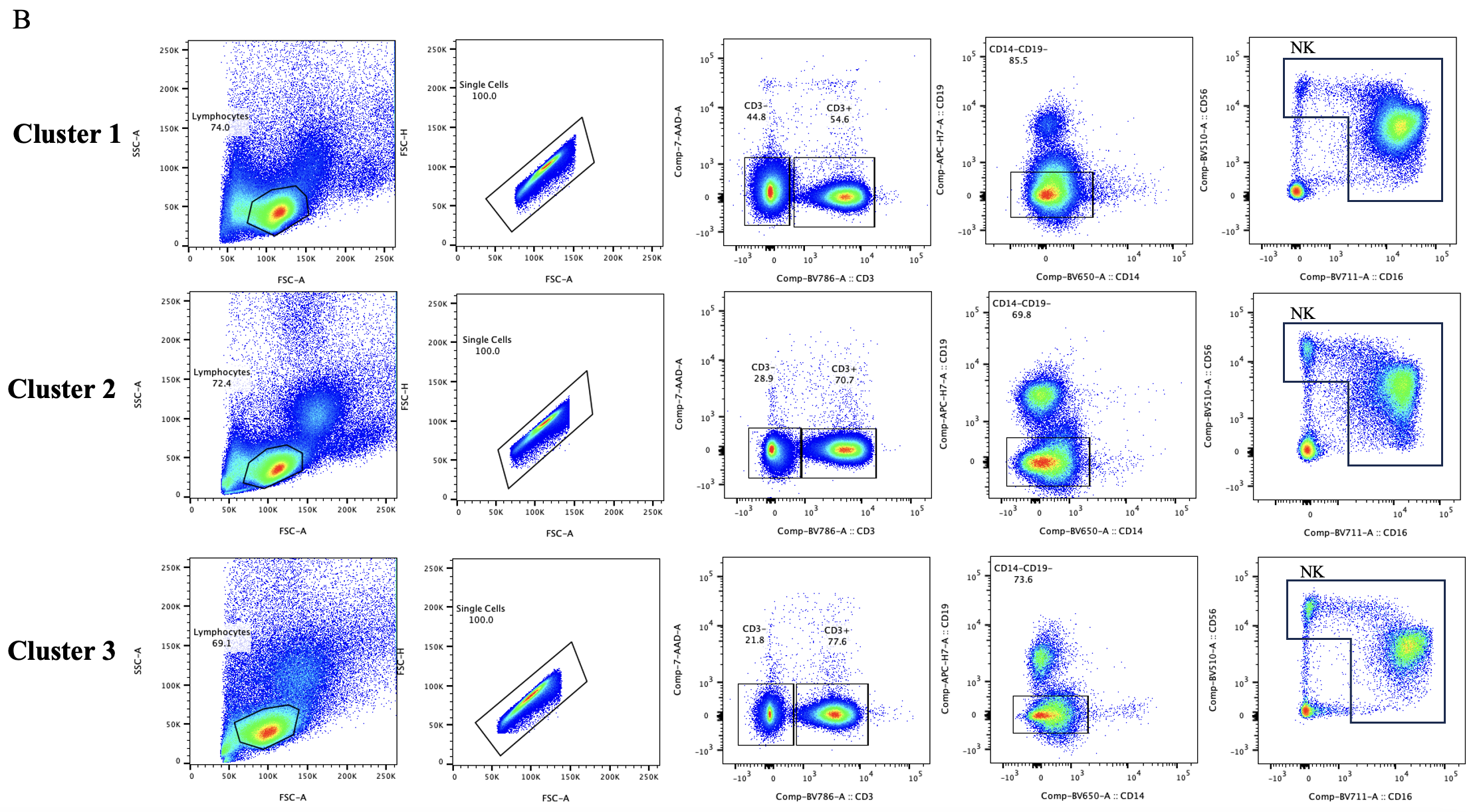


FigureS2. Gating strategy for T cell, NK cell flow cytometry and representative population expression proportion in systemic immune classification.


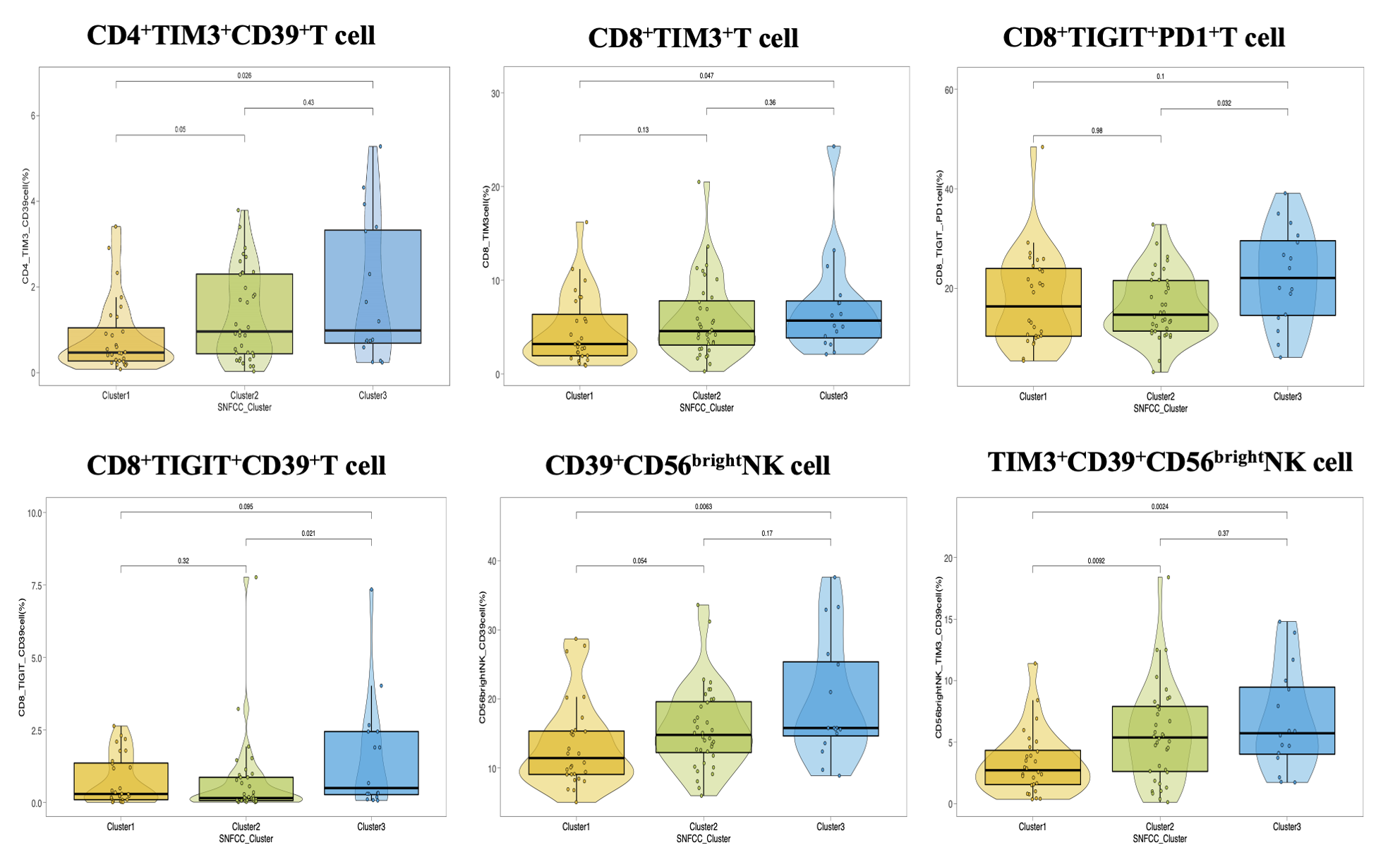

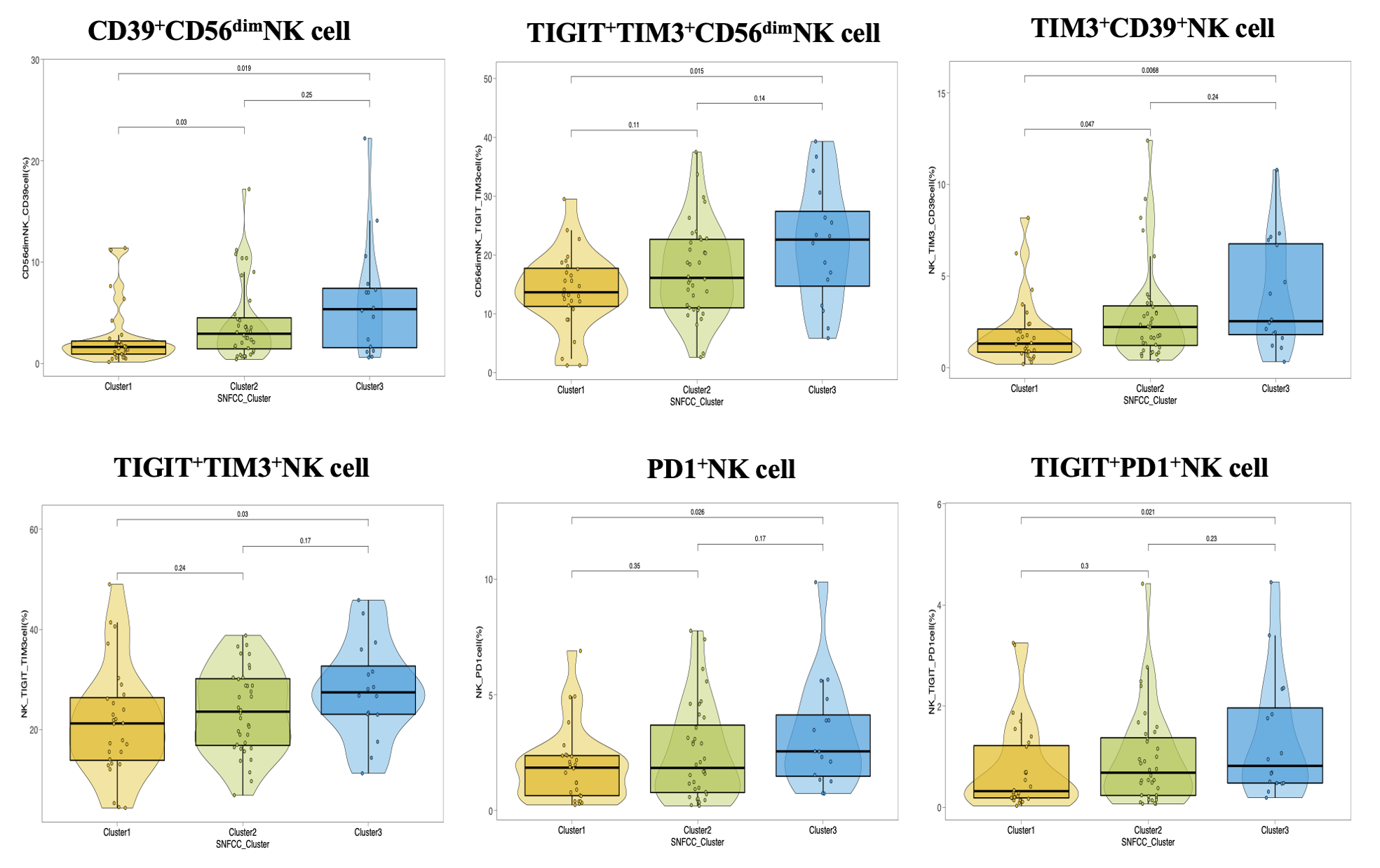


FigureS3. The phenotypes of immune cells exhaustion in HBV-HCC patients in systemic immune classification.


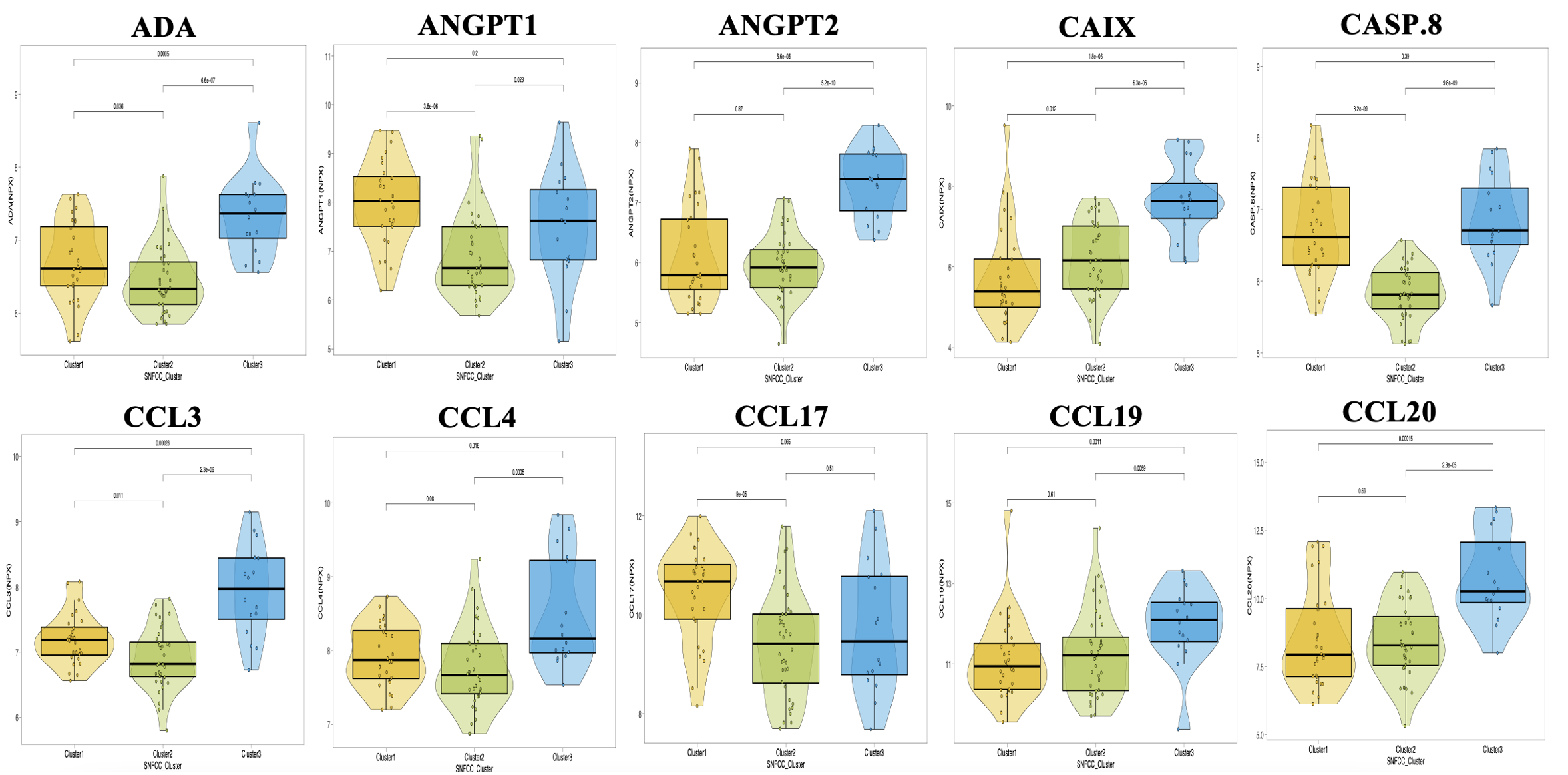

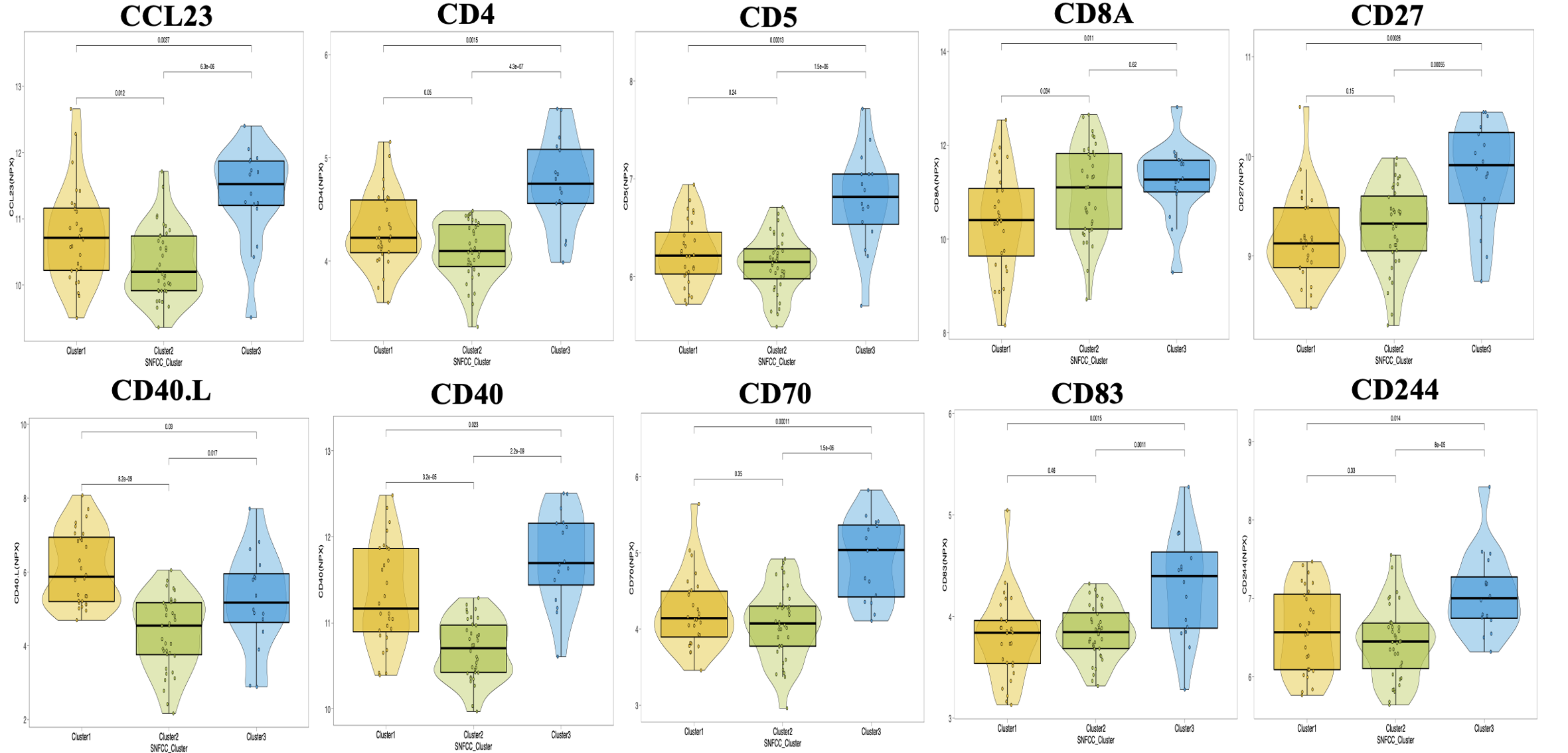


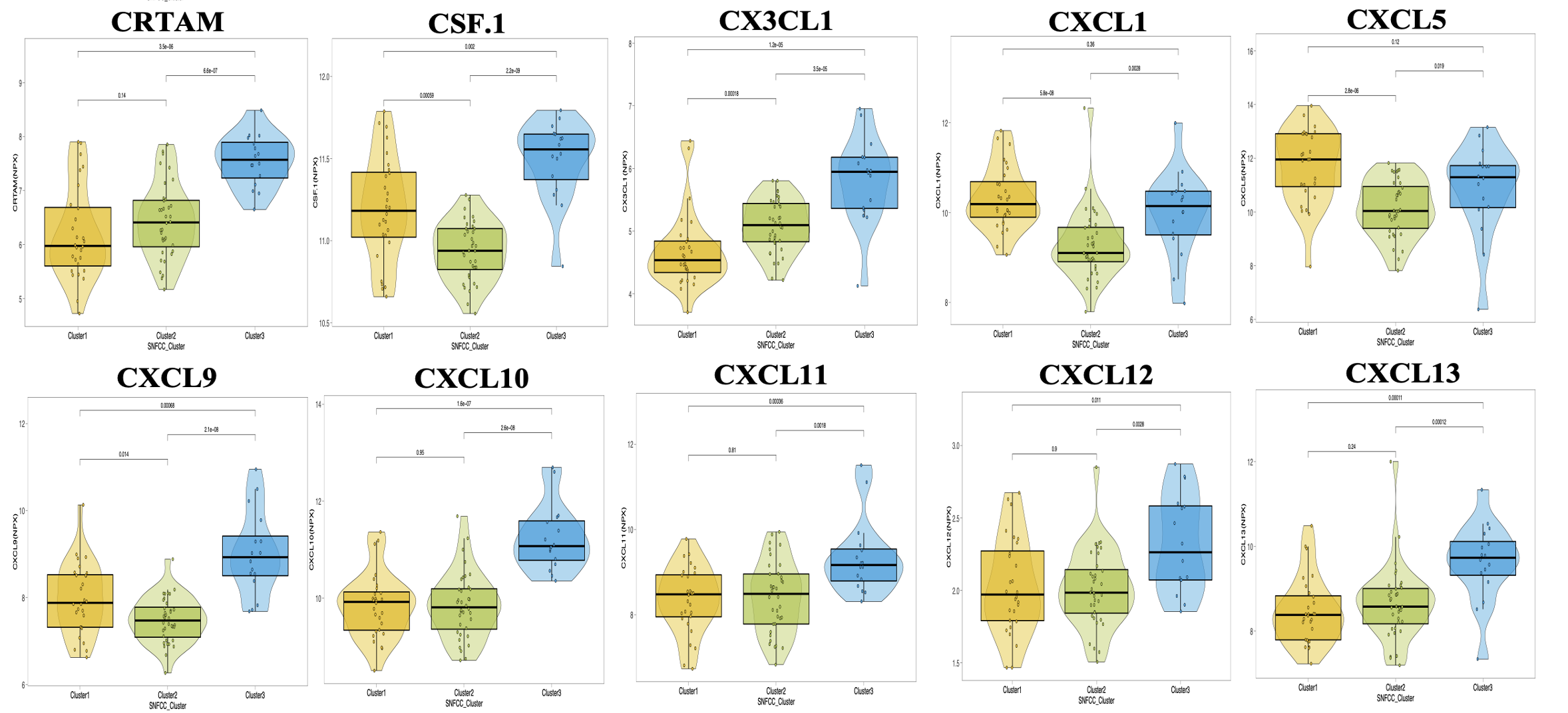

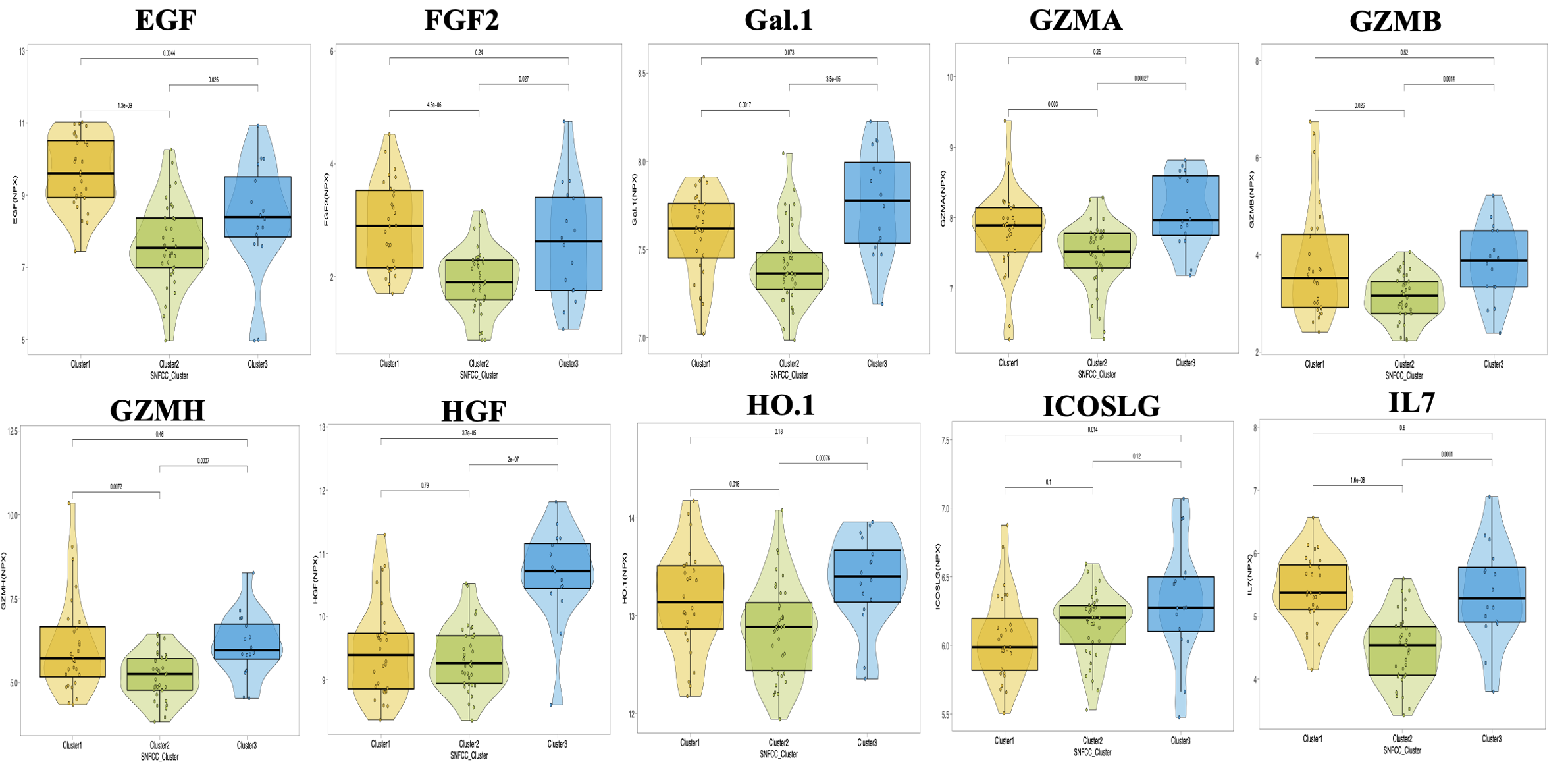


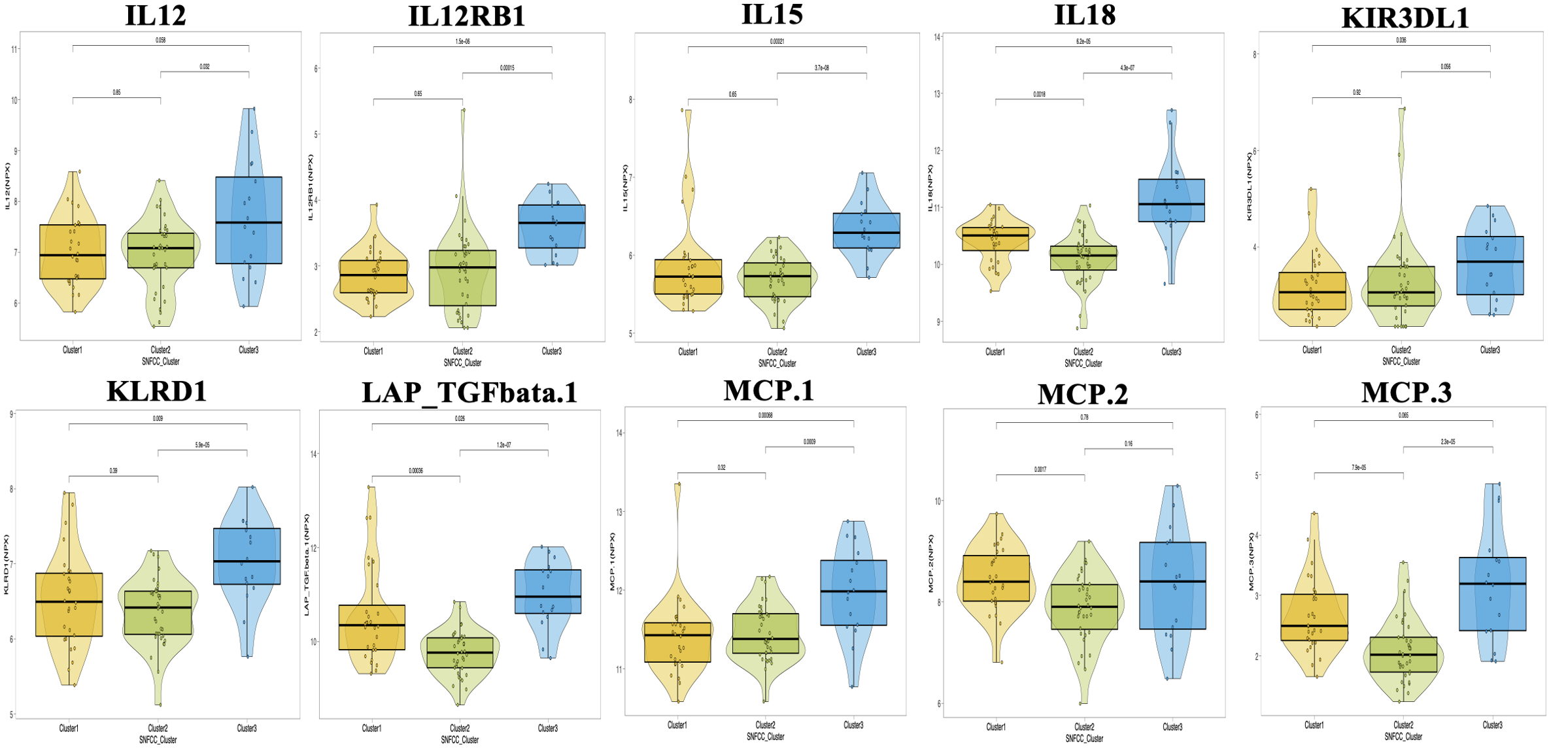

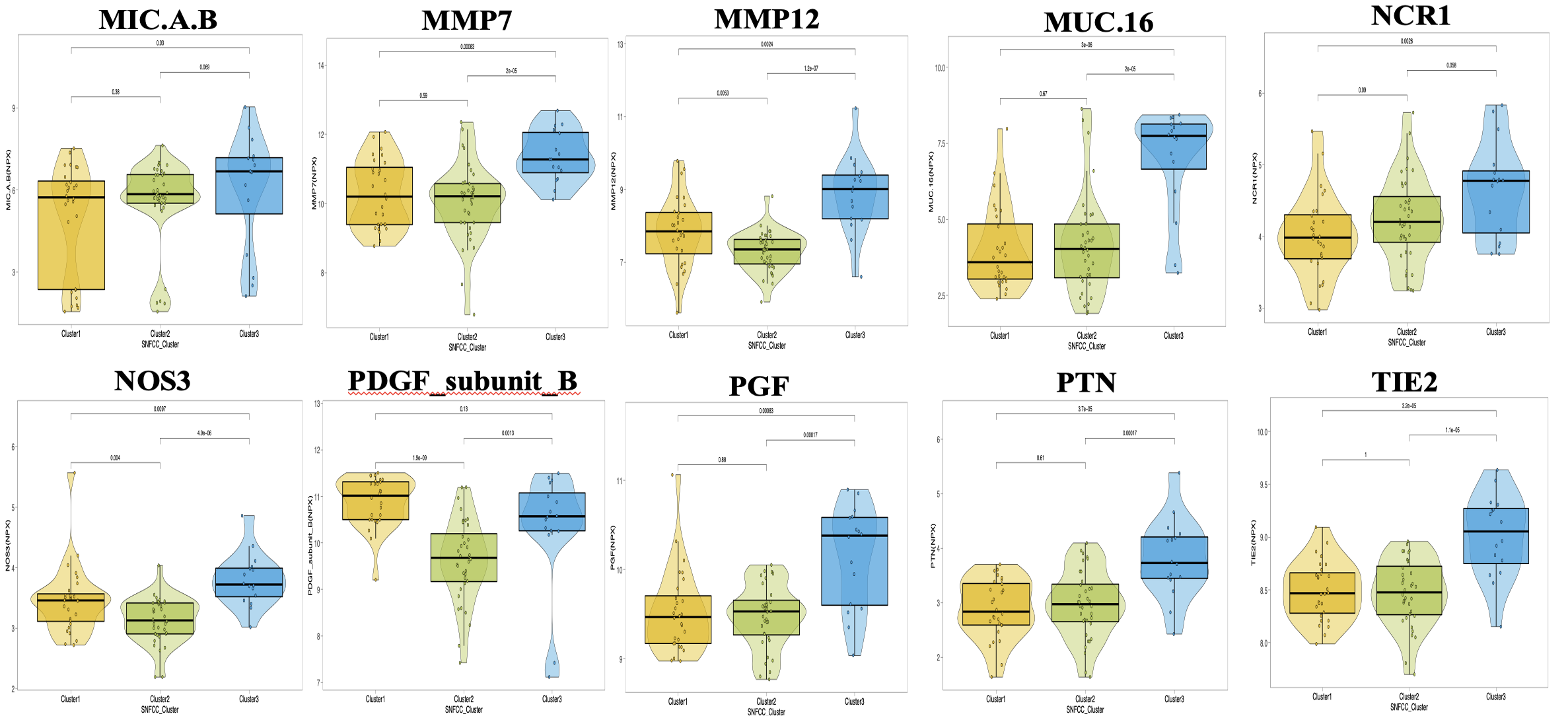

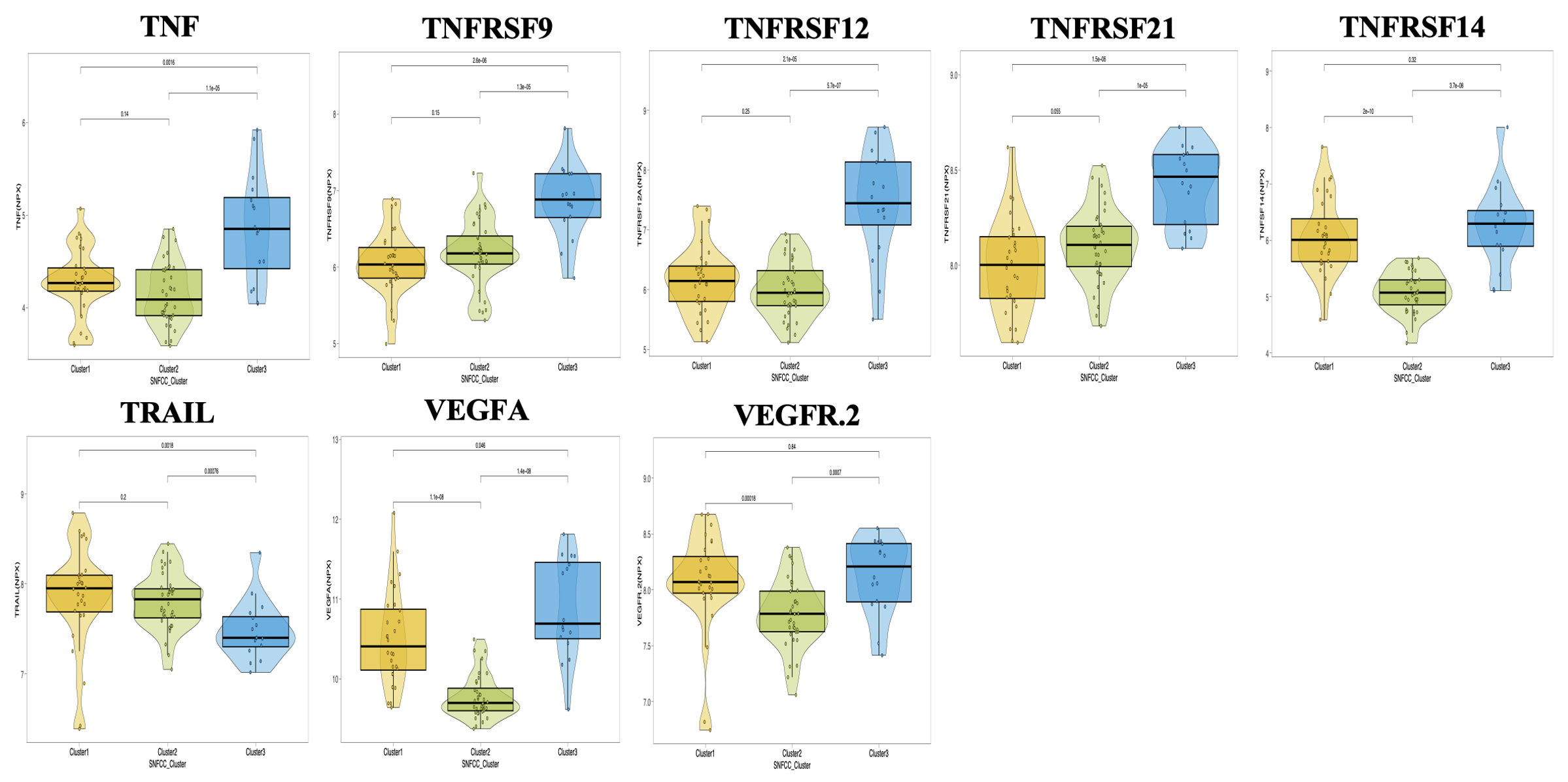


FigureS4. The phenotypes of immune proteins in HBV-HCC patients in systemic immune classification.


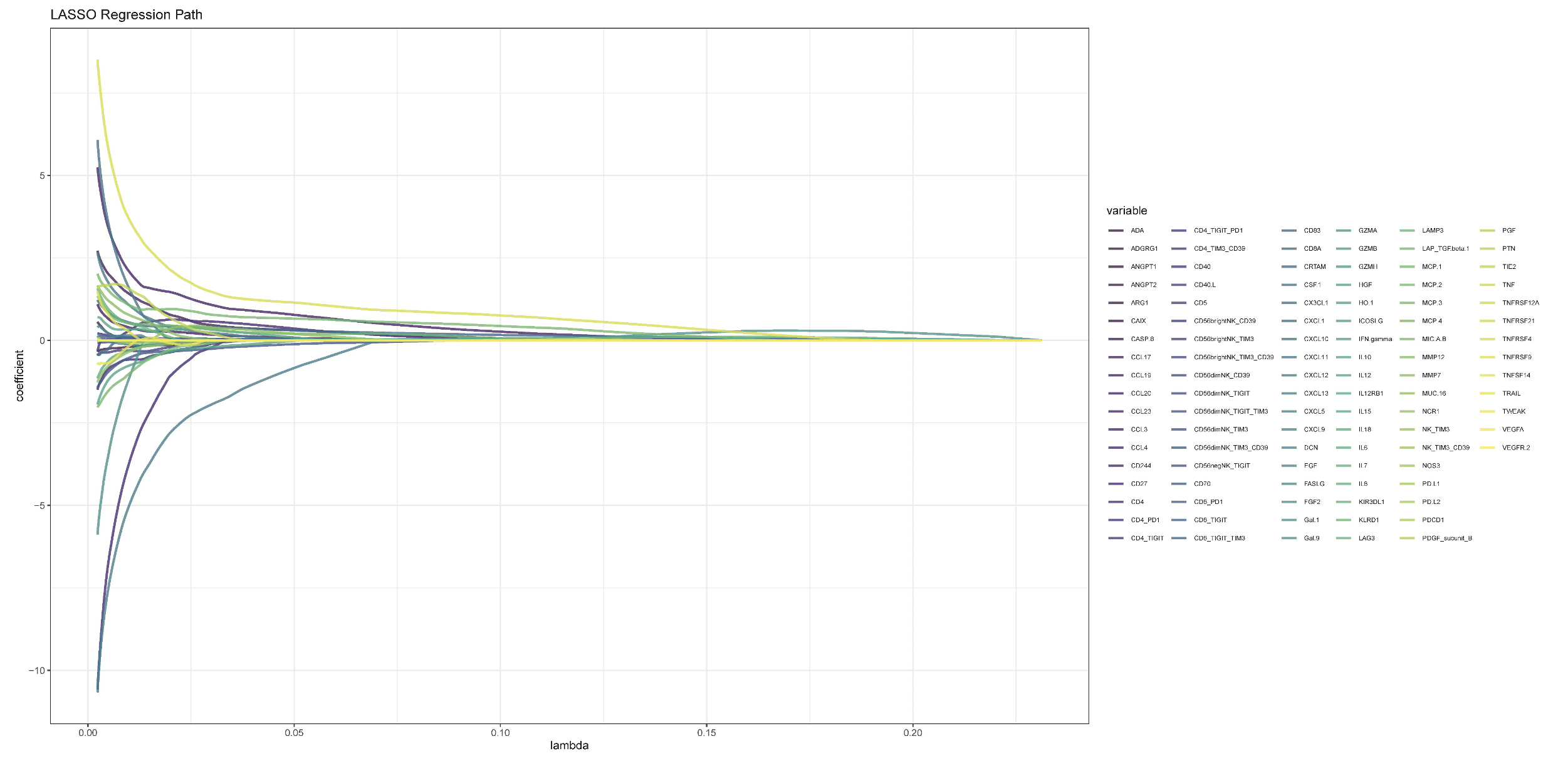


FigureS5. LASSO regression was used to construct a prognostic riskScore for HBV-HCC patients after TACE treatment.


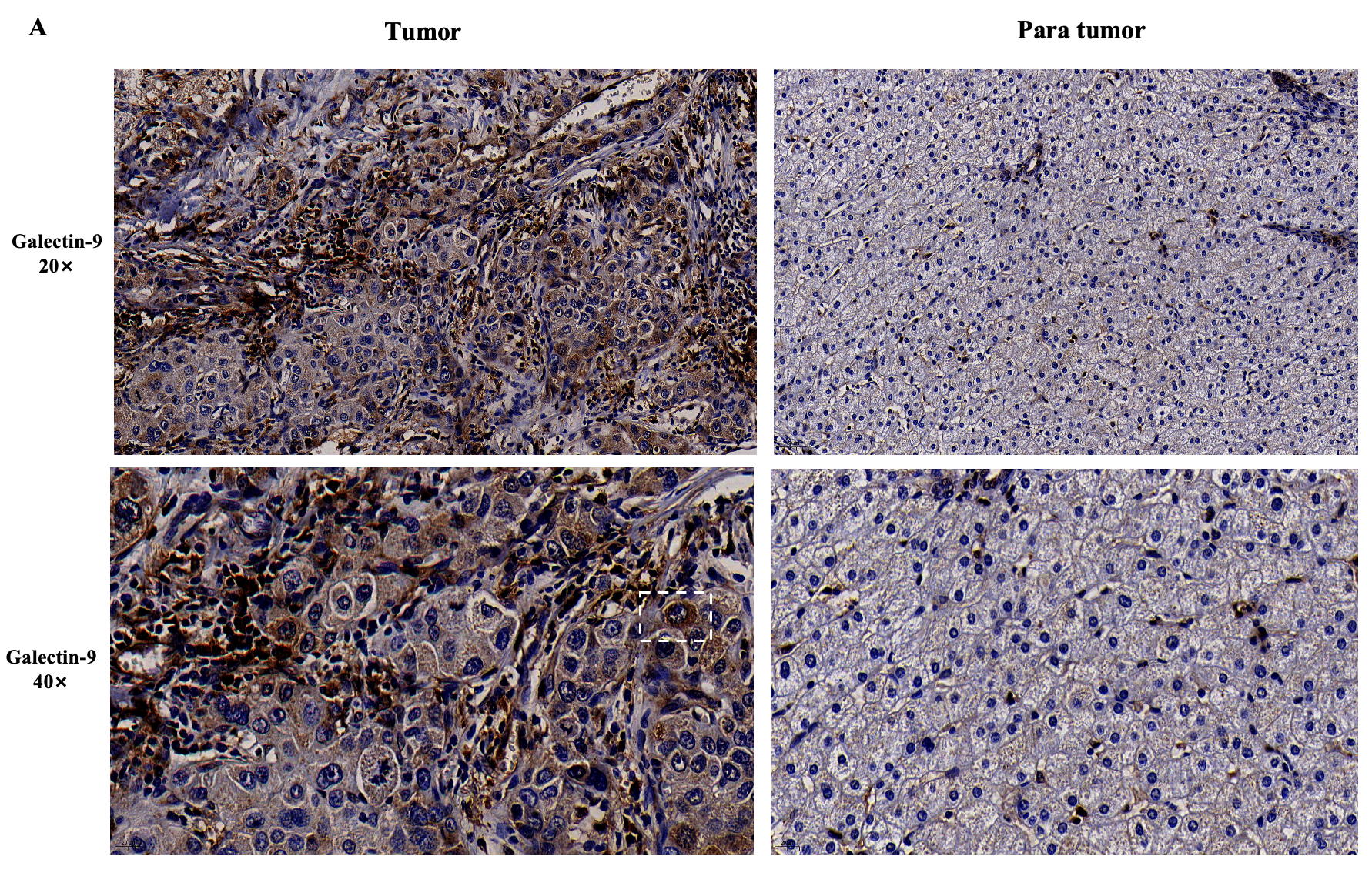

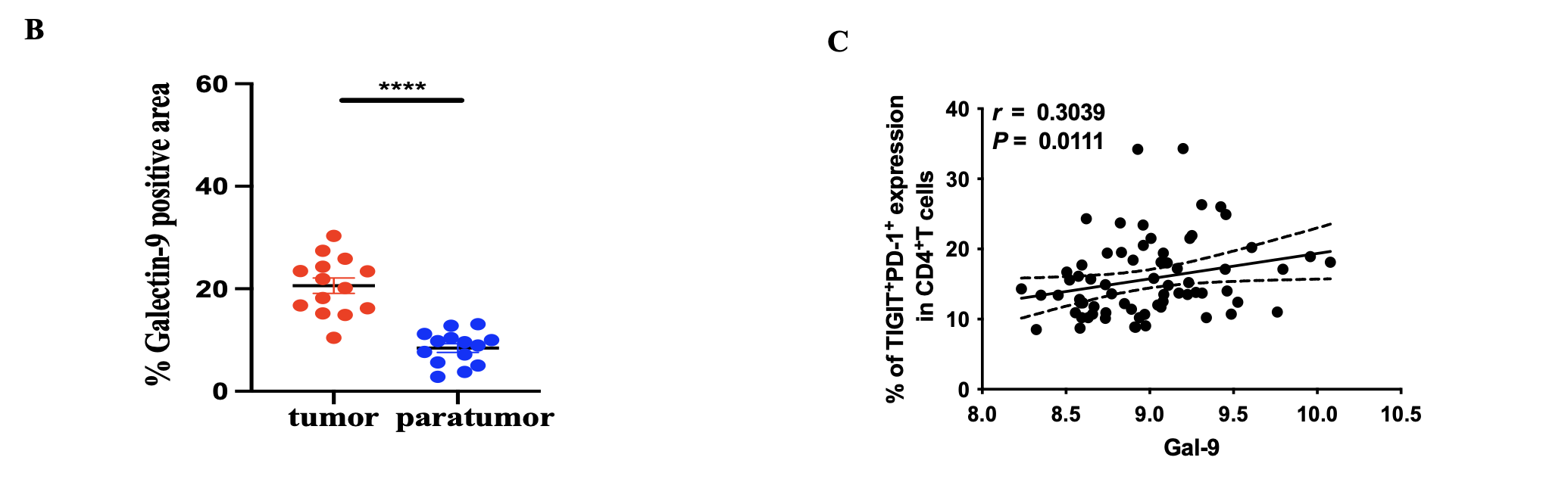


FigureS6. Characteristic analysis of Galectin-9 in HCC tissue. (A-B) Immunohistochemical staining of tumor tissue and adjacent tissues in patients after HCC resection. (C) The correlation between Galectin-9 and immune exhaustion phenotype. **P*<0.05, ***P*< 0.01, ****P*< 0.001, *****P*< 0.0001


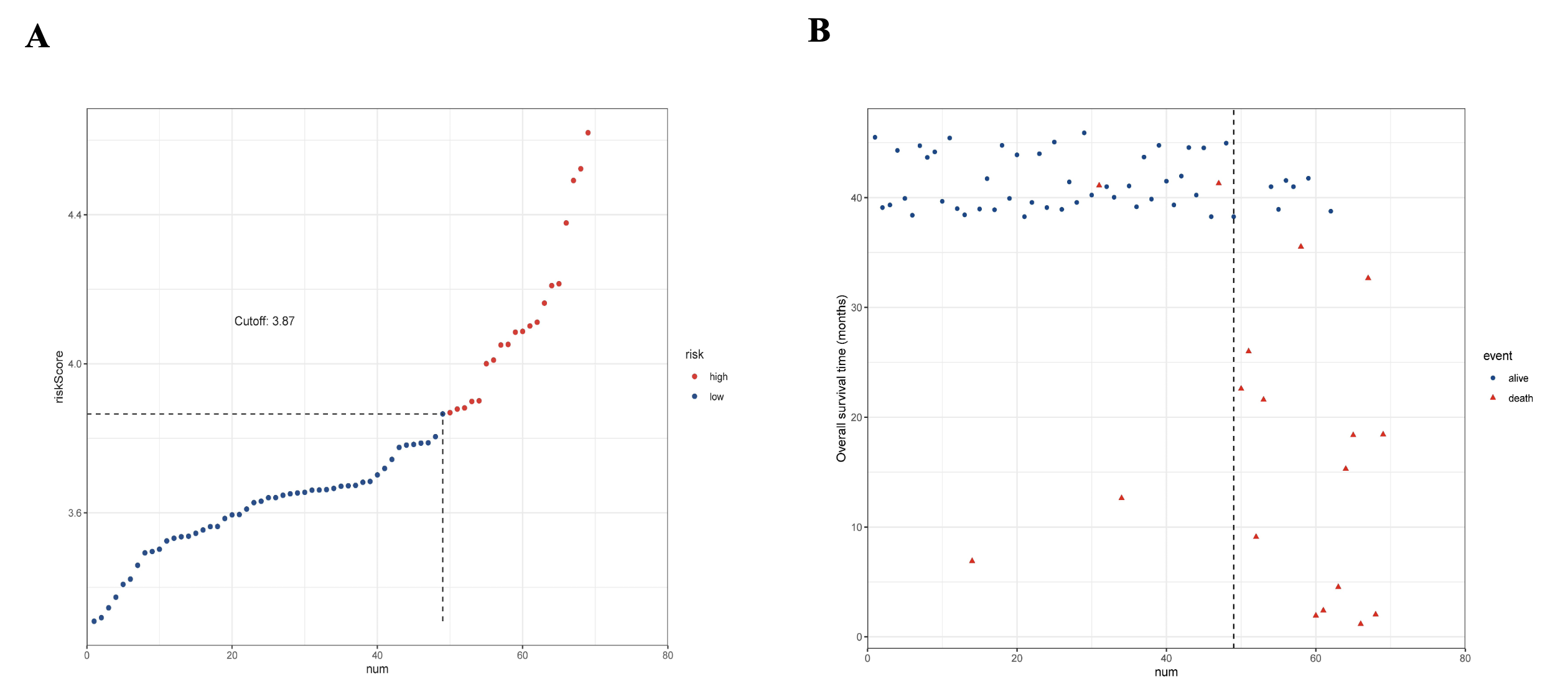


FigureS7. The prognostic riskScore for HBV-HCC patients after TACE treatment. (A) Cut-off value analysis of risk scoring. (B) Distributed in survival and death groups.


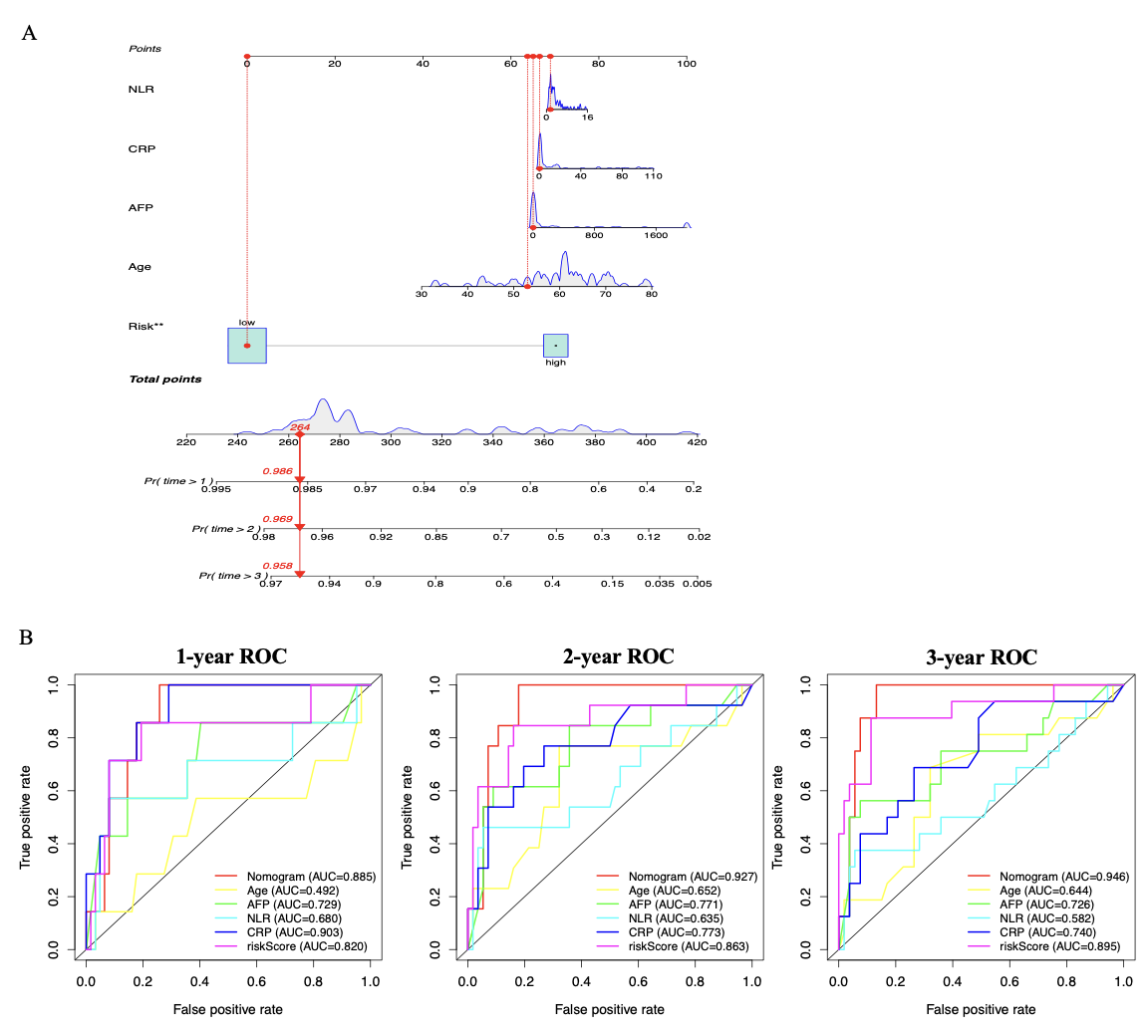


FigureS8. Establishing a nomogram model based on riskScore combined with clinical indicators to predict the OS of HBV-HCC patients after TACE treatment. (A)Nomogram model; (B) Comparison of ROC curves for nomogram models, risk scores, and other indicators.

**
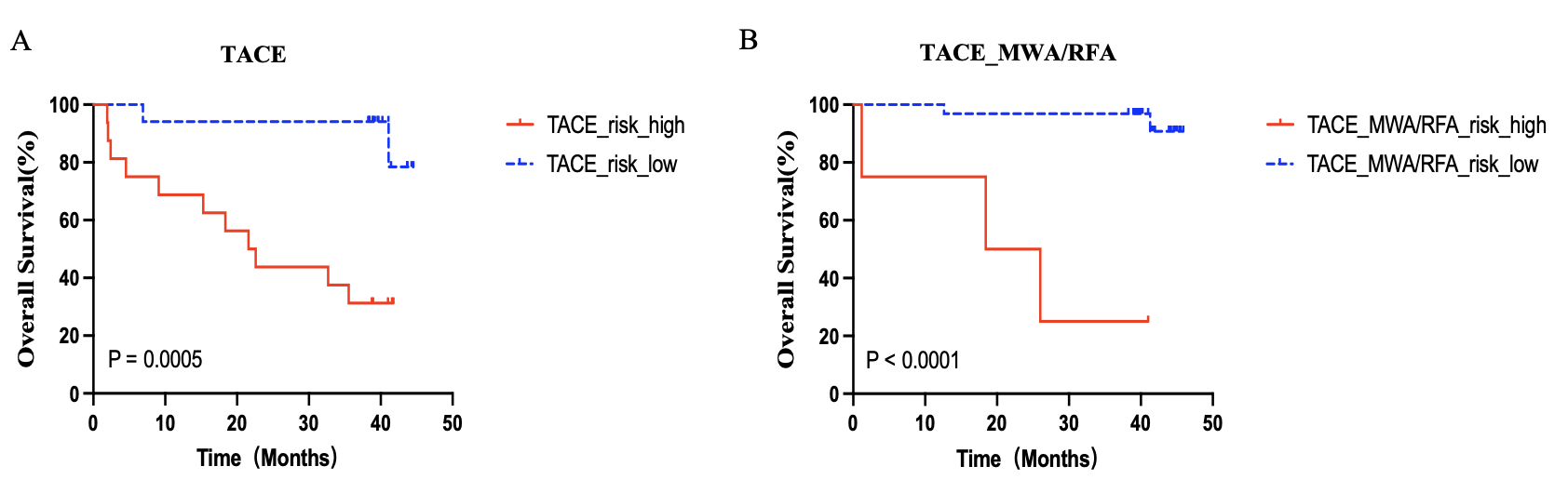
**

FigureS9. (A-B) Kaplan-Meier curve analysis was used to analyze the survival rate of HBV-HCC patients in different TACE alone and TACE combination therapy. **P*<0.05, ***P*< 0.01, ****P*< 0.001, *****P*< 0.0001


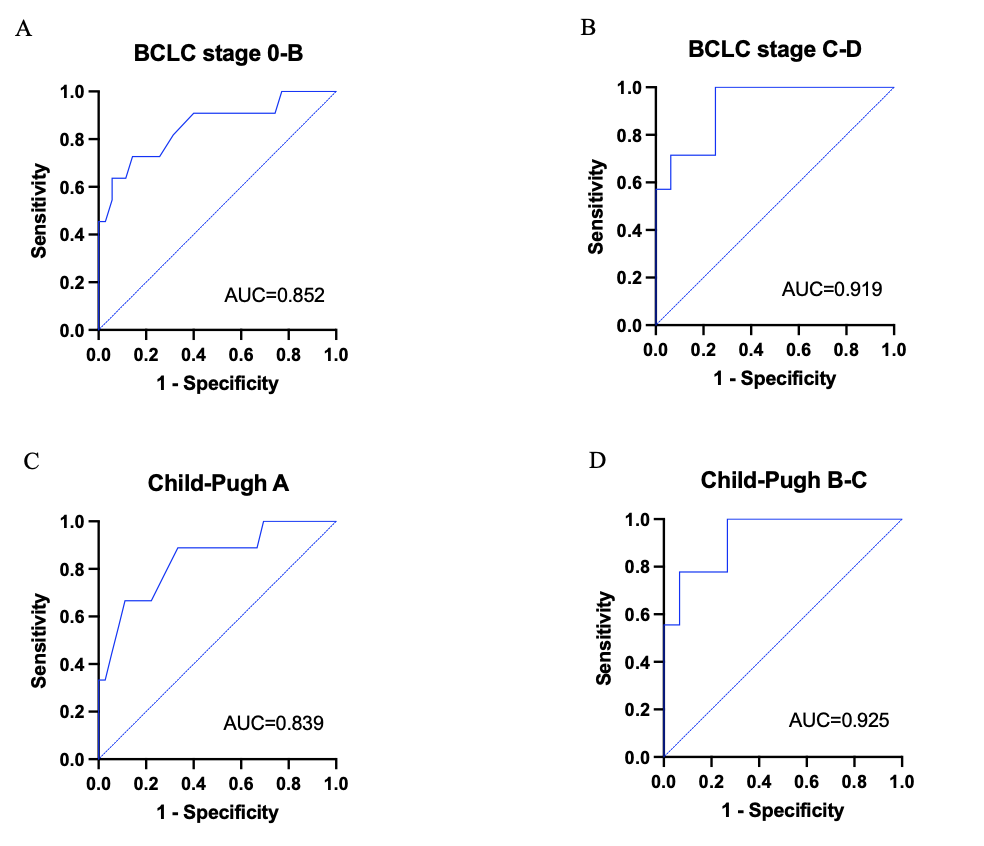


FigureS10. (A-B) Comparison of ROC curves for riskScore in BCLC stage 0-B and BCLC stage C-D. (C-D) Comparison of ROC curves for riskScore in Child-Pugh A and Child-Pugh B-C.
